# Iron homeostasis in *Arabidopsis thaliana*: transcriptomic analyses reveal novel FIT-regulated genes, iron deficiency marker genes and functional gene networks

**DOI:** 10.1186/s12870-016-0899-9

**Published:** 2016-10-03

**Authors:** Hans-Jörg Mai, Stéphanie Pateyron, Petra Bauer

**Affiliations:** 1 Institute of Botany, Heinrich Heine University Düsseldorf, Universitätsstraße 1, Building 26.13, 02.36, 40225 Düsseldorf, Germany; 2 Institute of Plant Sciences Paris Saclay IPS2, CNRS, INRA, Université Paris-Sud, Université Evry, Université Paris-Saclay, Bâtiment 630, 91405 Orsay, France; 3 Institute of Plant Sciences Paris-Saclay IPS2, Paris Diderot, Sorbonne Paris-Cité, Bâtiment 630, 91405 Orsay, France; 4 CEPLAS Cluster of Excellence on Plant Sciences, Heinrich Heine University Düsseldorf, Düsseldorf, Germany

**Keywords:** Plants, Arabidopsis, Iron homeostasis, FIT, Differential gene expression, Microarray

## Abstract

**Background:**

FIT (FER-LIKE IRON DEFICIENCY-INDUCED TRANSCRIPTION FACTOR) is the central regulator of iron uptake in *Arabidopsis thaliana* roots. We performed transcriptome analyses of six day-old seedlings and roots of six week-old plants using wild type, a *fit* knock-out mutant and a FIT over-expression line grown under iron-sufficient or iron-deficient conditions. We compared genes regulated in a FIT-dependent manner depending on the developmental stage of the plants. We assembled a high likelihood dataset which we used to perform co-expression and functional analysis of the most stably iron deficiency-induced genes.

**Results:**

448 genes were found FIT-regulated. Out of these, 34 genes were robustly FIT-regulated in root and seedling samples and included 13 novel FIT-dependent genes. Three hundred thirty-one genes showed differential regulation in response to the presence and absence of FIT only in the root samples, while this was the case for 83 genes in the seedling samples. We assembled a virtual dataset of iron-regulated genes based on a total of 14 transcriptomic analyses of iron-deficient and iron-sufficient wild-type plants to pinpoint the best marker genes for iron deficiency and analyzed this dataset in depth. Co-expression analysis of this dataset revealed 13 distinct regulons part of which predominantly contained functionally related genes.

**Conclusions:**

We could enlarge the list of FIT-dependent genes and discriminate between genes that are robustly FIT-regulated in roots and seedlings or only in one of those. FIT-regulated genes were mostly induced, few of them were repressed by FIT. With the analysis of a virtual dataset we could filter out and pinpoint new candidates among the most reliable marker genes for iron deficiency. Moreover, co-expression and functional analysis of this virtual dataset revealed iron deficiency-induced and functionally distinct regulons.

**Electronic supplementary material:**

The online version of this article (doi:10.1186/s12870-016-0899-9) contains supplementary material, which is available to authorized users.

## Background

Iron is an essential micronutrient for plants but excess iron can be toxic. Hence, plant iron homeostasis is tightly regulated. Strategy I plants take up reduced iron. First, the rhizosphere is acidified by proton extrusion to solubilize Fe^3+^, then Fe^3+^ is reduced to Fe^2+^ which is finally taken up into the root epidermis cell by a specific transporter [1–3]. In *Arabidopsis thaliana*, belonging to the group of Strategy I plants, the P-type H^+^-ATPase AHA2 extrudes protons into the rhizosphere [4]. Ferric iron is reduced by the ferric chelate reductase FRO2 (FERRIC REDUCTION OXIDASE 2) which is induced by iron deficiency in the root epidermis [5, 6]. Finally, ferrous iron is translocated into the root cell by IRT1 (IRON-REGULATED TRANSPORTER 1) [7–10]. Expression of *AHA2*, *FRO2* and *IRT1* is regulated by FIT (FER-LIKE IRON DEFICIENCY-INDUCED TRANSCRIPTION FACTOR) [11–14]. Even under strong constitutive FIT expression *IRT1* and *FRO2* are induced only under iron deficient conditions [11] and ectopic expression of *IRT1* and *FRO2* in leaves only occurs under iron deficiency [12]. Hence, FIT activity is post-translationally regulated [12], and FIT protein-protein interactions have been found that can explain this behavior [15–17]. Loss-of-function mutants of *fit* exhibit symptoms of iron starvation like chlorosis, reduced growth and lethality [11, 12, 18].

To gain better understanding of the gene regulatory processes transcriptomic analyses with regard to iron homeostasis in *A. thaliana* have been performed with diverse results. Iron deficiency causes activation of distinct functional modules such as the ‘transportome’ which, among others, includes genes that are involved in transition metal detoxification [19]. Ethylene signaling-related genes and a number of iron-responsive genes are expressed in an ethylene-dependent manner such as *FIT*, *IRT1*, *NAS1*, *NAS2*, *FRD3* and the gene of a 2OG-Fe(II) oxygenase family protein [20]. EIN3/EIL1 appear to be required for reorganization of the photosystems to reduce photo-oxidative damage and this could also be achieved under iron deficiency by EIN3/EIL1-mediated increase of iron uptake [16]. Copper deficiency causes secondary iron deficiency in Arabidopsis and leads to up-regulation of *IRT1* and *FRO2* [21]. There is crosstalk between copper and iron uptake and phosphate starvation and there are indications for different functions of copper under iron deficiency and phosphate starvation [22]. microRNAs were demonstrated to negatively regulate CuSOD (copper containing superoxide dismutase) genes allowing increased CuSOD expression to functionally replace FeSODs (iron containing superoxide dismutases) under iron deficiency in *A. thaliana* rosette leaves [23]. Time course transcriptomic analyses showed that distinct sets of genes are up- and down-regulated at different time points after induction of iron deficiency [24]. Another time course experiment revealed that PYE (POPEYE) is involved in iron homeostasis by regulating genes such as *FER1*, *FER4*, *OPT3*, *NAS4*, *FRO3*, *NRAMP4* and *FRD3* [25].

Based on results of many microarray analyses, the creation and analysis of co-regulatory networks of iron-responsive genes have gained increasing interest. Prominent publicly available tools for such network analyses are ATTED II [26] and STRING [27]. Co-expression and interaction network analyses may help identify further important genes as potential targets of future investigations and hence contribute to discover new aspects of the plant’s physiological reaction to iron deficiency and the respective underlying control mechanisms. For example, co-expression analyses revealed multiple subnetworks for iron homeostasis functions including the PYE-BTS regulon [25] and iron uptake including FIT targets like *IRT1*. Some of these genes are robust markers for iron deficiency in *A. thaliana* roots [13].

So far, there are only few known marker genes for iron deficiency [13]. A number of FIT-dependent genes have been determined in a previous study using the *fit-1* mutant [14] which is a promoter T-DNA insertion line with residual *FIT* expression [11]. Furthermore, this study has been performed using ATH1 Affymetrix chips which to this date lack a number of genes including important iron homeostasis-related genes such as *FIT* and *FRO2*. It can be speculated that *fit1-1* plants display a rather intermediate reaction to iron deficiency due to their residual FIT expression and that not all FIT-dependent genes could have been detected due to the use of ATH1 Affymetrix chips. So far, no FIT over-expression line has been employed in the search for FIT-dependent genes which might contribute to refinement of the search results. Furthermore, it has not yet been investigated whether the developmental stage of the plants influences the dependence of genes on FIT. To address these questions, we conducted transcriptomic analyses of roots of six week-old plants and six day-old seedlings that were exposed to iron-deficient or iron-sufficient conditions using the *Arabidopsis thaliana* Col-0 (wild type), *fit-3* (exon T-DNA insertion *fit* knock-out) [12] and HA-FIT (*pCaMV35S::HA*_*7*_*-FIT*) over-expression lines [28]. We stringently filtered the genes by their expression patterns to obtain a comprehensive list of known and novel FIT-dependent genes. This same filtering process was then used to determine genes that were affected by the presence of FIT only in roots or seedlings, respectively. Furthermore, we assembled a virtual dataset based on our gene expression data plus previous transcriptomic analyses to pinpoint more reliably iron deficiency-regulated marker genes and used this dataset to perform co-expression analysis.

## Results and discussion

### Overview of the microarray analyses

A number of genes that are potentially regulated by FIT have been pointed out by Colangelo and Guerinot [11]. Since the study has been performed with wild-type Col-0 and the *fit-1* knock-out mutant [11, 14] which is a promotor T-DNA line, we decided to extend the analytic strategy by using a *fit* knock-out mutant with the strong *fit-3* allele which is an exon T-DNA knock-out mutant [12, 14] (hereafter termed *fit*) and by including the *FIT*-overexpression line HA-FIT 8 (hereafter called HA-FIT) [28] to define a full set of genes that are regulated by FIT. We first analyzed the transcriptomic changes in roots of six week-old wild type, *fit* and HA-FIT plants that were exposed to iron-sufficient (+Fe) or iron-deficient (-Fe) conditions for 7 days prior to harvesting and the same analyses were conducted with six-day-old whole seedlings that were grown on  +Fe or -Fe (Additional file 1: Figure S1). Using CATMA two-color microarrays we performed seven pairwise comparisons with three biological and two technical replicates, respectively. In three pairwise comparisons we measured the transcriptomic changes upon iron deficiency within the lines. These include wild type -Fe vs. +Fe, *fit* -Fe vs. +Fe and HA-FIT -Fe vs. +Fe. We refer to these comparisons as ‘intra-line comparisons’. The four other pairwise comparisons monitor transcriptomic differences between the lines at a given iron status. They include the comparisons *fit* vs. wild type and HA-FIT vs. wild type both at  +Fe and -Fe, respectively. We refer to them as ‘inter-line comparisons’. To validate the seedling data, we performed RT-qPCR with selected iron homeostasis-related genes (Additional file 2: Figure S2). The root data were previously validated by [29].

### Gene regulation in roots from six-week-old Arabidopsis plants

**Fig. 1:**
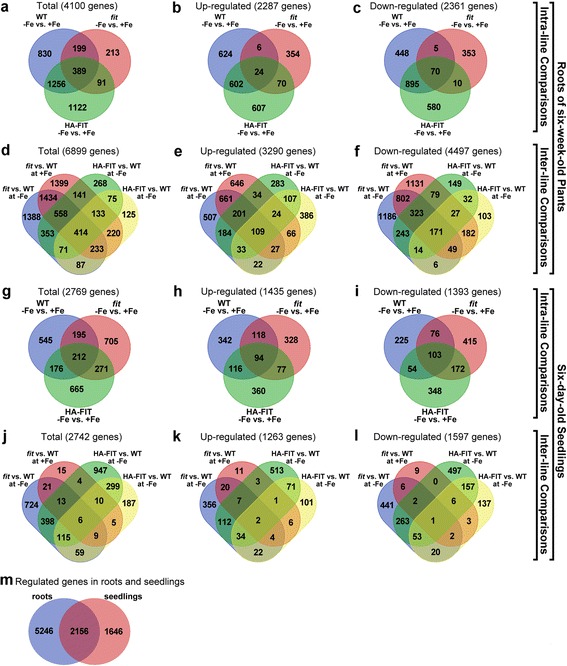
Venn diagrams of the differentially expressed genes in the six-week-old Arabidopsis roots (**a**-**f**) and six-day-old seedlings (**g**-**l**). Intra-line comparisons in roots (**a**-**c**): Total numbers of regulated genes (**a**), induced genes (**b**) and repressed genes (**c**). Inter-line comparisons in roots (**d**-**f**): Total numbers of regulated genes (**d**), induced genes (**e**) and repressed genes (**f**). Intra-line comparisons in seedlings (**g**-**i**): Total numbers of regulated genes (**g**), induced genes (**h**) and repressed genes (**i**). Inter-line comparisons in seedlings (**j**-**l**): Total numbers of regulated genes (**j**), induced genes (**k**) and repressed genes (**l**). Intersection between regulated genes in roots and seedlings (**m**). The diagrams were created using the unnamed online tool provided by VIB/U Gent, Bioinformatics & Systems Biology, Technologiepark 927, B-9052 Gent, Belgium; accessible through http://bioinformatics.psb.ugent.be/webtools/Venn/

First, when gene expression in roots of six-week-old wild type plants was analyzed, a total number of 7402 genes was found regulated in at least one out of the seven comparisons (Additional file 3: Dataset 1). Four thousand one hundred genes were found regulated in the intra-line comparisons (Fig. 1a). Out of these, 2287 were up-regulated (Fig. 1b) and 2361 were down-regulated (Fig. 1c) at -Fe in at least one of the comparisons. The least number of regulated genes at  +Fe versus -Fe was found in *fit*. Four hundred fifty-four genes were induced and 438 genes repressed in *fit*. The number of induced and repressed genes in wild type was 1256 and 1418, respectively, while in HA-FIT 1303 genes were induced and 1555 genes repressed under -Fe. The less pronounced transcriptomic reaction to -Fe in *fit* can be explained by the fact that *fit* plants suffered from iron deficiency although they were grown on iron-sufficient medium. Hence, the primary and secondary adaptations to -Fe that can be observed in wild type and HA-FIT may have largely been established in *fit* plants under  +Fe already. Additionally, the lack of FIT may cause the inability to induce or repress a subset of genes as soon as further iron deficiency is sensed. The little overlaps between *fit* and the other lines and the large overlaps between wild type and HA-FIT (Fig. 1b and Fig. 1c) also suggest that six-week-old *fit* roots react in a more distinct way to iron deficiency while wild type and HA-FIT show similar responses.

In the inter-line comparisons a total of 6899 genes were found regulated (Fig. 1d). More genes were regulated in the inter-line comparisons (Fig. 1d) than in the intra-line comparisons (Fig. 1a). Hence, differential gene expression between the different lines at a given iron supply is larger than the reaction of the respective lines to iron deficiency. Out of the 6899 regulated genes in the inter-line comparisons 3290 were up-regulated (Fig. 1e) and 4497 were down-regulated (Fig. 1f). Thirteen percent (888 genes) were found up- and down-regulated in different lines suggesting a regulation by FIT. Whereas the number of regulated genes was lower in *fit* under -Fe than in the other lines, the transcriptomic changes between *fit* and wild type were much greater than between HA-FIT and wild type. In conclusion the transcriptomes of HA-FIT and wild type are more similar to each other while six-week-old *fit* plants display distinct transcriptomic adaptations.

### Gene regulation in 6-day-old Arabidopsis seedlings

In six-day-old seedlings 3802 genes were found regulated in at least one of the seven comparisons (Additional file 3: Dataset 2). With 2769 genes the number of regulated genes in the inter-line comparisons of six-day-old seedlings (Fig. 1g) is 32 % less than in roots of six week-old plants. Out of these, 1435 genes were found induced (Fig. 1h) and 1393 genes were found repressed (Fig. 1i) under -Fe. The overlap between the up- and down-regulated genes was only 2 % (59 genes). This suggests that gene regulation upon iron deficiency is very specific to the investigated lines. In contrast to the six-week-old roots we could not observe a large difference in the number of regulated genes that overlap between *fit*, wild type and HA-FIT seedlings in the intra-line comparisons. All three lines shared roughly similar numbers of regulated genes, being reduced or repressed. The number of genes regulated in seedlings of the single lines did not differ as much as in roots of six-week-old plants. Therefore, seedlings of the three lines react more similarly to iron deficiency than roots of adult plants.

In the inter-line comparisons 2742 genes were found regulated (Fig. 1j). Out of these, 1263 genes were induced (Fig. 1k) and 1597 genes were repressed (Fig. 1l) in at least one of the comparisons. The intersection between up- and down-regulated genes was 4 % (118 genes). This again suggests that the observed regulations were predominantly specific to the compared lines. In contrast to the six-week-old roots where the most pronounced regulation in the intra-line comparisons was found in *fit* this could not be observed in the six-day-old seedlings. Five hundred fifty-seven genes were induced (Fig. 1k) and 788 were repressed under iron-deficient conditions in *fit* seedlings compared to wild type (Fig. 1l) under -Fe. Only 54 genes were induced (Fig. 1k) and 29 genes were repressed under  +Fe in *fit* seedlings compared to wild type (Fig. 1l). From the lower numbers of regulated genes and the higher line and comparison-specific gene regulation we conclude that the six-day-old seedlings display a transcriptomic reaction that is distinct from the differential gene expression in six-week-old roots at the large scale.

Taken together, the total number of regulated genes was larger in the roots of six-week-old plants than in seedlings (Fig. 1m). Seedlings may programmed to quickly increase their biomass and uptake and utilization of nutrients may be generally enhanced, which could lead to a less responsive gene regulation upon iron deficiency. Iron is also stored in vacuoles of the embryo [30–32] so there is a pool of usable iron which might contribute to a less pronounced transcriptomic reaction to iron-deficient medium in young seedlings. Another possible explanation could be that older roots are fully differentiated and react differently and more intensively to iron deficient conditions. However, among the 7402 and 3802 genes regulated in roots and seedlings, respectively, there is a comparably large intersection of 2156 genes which still points towards a certain common transcriptomic reaction in roots and seedlings (Fig. 1m). Furthermore, the numbers of regulated genes show that the influence of FIT on gene expression is higher than the impact of iron deficiency alone. In roots of HA-FIT and wild type more genes were regulated in -Fe versus  +Fe than in *fit* and the intersection between HA-FIT and wild type was larger than between *fit* and the two other lines. This is in agreement with the fact that *fit* mutants cannot react to iron deficiency as wild type or the *FIT* over-expressor.

### Hierarchical clustering of the microarray results in iron homeostasis-enriched gene clusters

To detect regulatory patterns we performed hierarchical clustering with the datasets from the six-week-old Arabidopsis roots (Additional file 3: Dataset 1), the six-day-old seedlings (Additional file 3: Dataset 2) and the combination of both (Additional file 3: Dataset 3). We highlighted clusters in which the two confirmed FIT-regulated marker genes *IRT1* and *FRO2* [12], *AT3G13610*, *AT3G07720*, *MTPA2* and *COPT2* [11] were present.

**Fig. 2:**
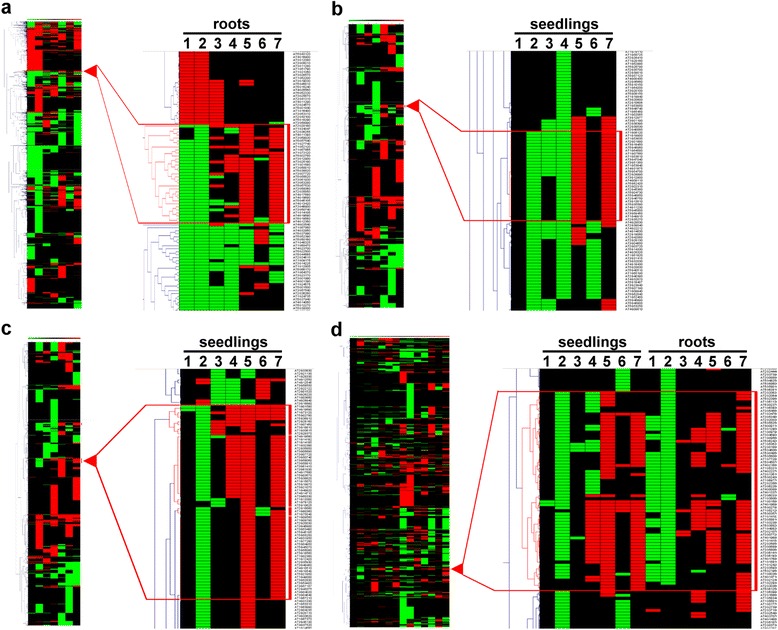
Hierarchical clustering of the genes that were differentially regulated in six-day-old seedlings (**a** and **b**). Roots of six-week-old plants (**c**) and in the combined analysis (**d**). The respectively compared lines and conditions are indicated by numbers. 1: *fit* +Fe vs. WT  +Fe. 2: *fit* -Fe vs. WT -Fe. 3: HA-FIT  +Fe vs. WT  +Fe. 4: HA-FIT -Fe vs. WT -Fe. 5: HA-FIT -Fe vs. HA-FIT  +Fe. 6: WT -Fe vs. WT  +Fe. 7: *fit* -Fe vs. *fit*  +Fe. The left panels show an overview over the whole cluster analysis and the right panel shows a magnified view of the respective cluster that is indicated by the red triangle and that contains known iron homeostasis-related genes. Red color represents up-regulation and green color represents down-regulation. The cluster analysis has been performed with Genesis [76]

In the dataset generated from the six-day-old seedlings the FIT-regulated marker genes appeared in five clusters of which four were directly adjacent and contained a total of 65 genes (Fig. 2a). The fifth cluster was distinct from the others and contained 30 genes (Fig. 2b). In the dataset from the six-week-old roots, the marker genes were found in one cluster containing 33 genes (Fig. 2c). Finally, in the combined dataset of both roots and seedlings, the marker genes were found in a single cluster containing 65 genes (Fig. 2d, Additional file 4: Figure S3). When focusing on the genes that clustered with FIT-regulated marker genes, 14 genes were found co-regulated in a robust manner in three clusters. Thirty-three genes were found in two of three clusters and 85 genes were present in only one out of the three clusters (Additional file 5: Table S1). Among the genes that were found in the clusters there were 38 previously FIT-associated ones. Ten of these were present in all three clusters, 16 in two of three clusters and 12 in one of the three clusters (Additional file 5: Table S1).

### Stringent expression pattern filtering revealed robustly FIT-dependent genes

To detect novel FIT targets, we performed stringent filtering of the 9048 regulated genes in the combined dataset of roots and seedlings (Additional file 3: Dataset 3). At first, we reduced the list of all genes to those genes that were found regulated in at least one comparison in seedlings and roots, thereby reducing the number to 2156 genes (intersection in Fig. 1m, Additional file 3: Dataset 4).

**Fig. 3:**
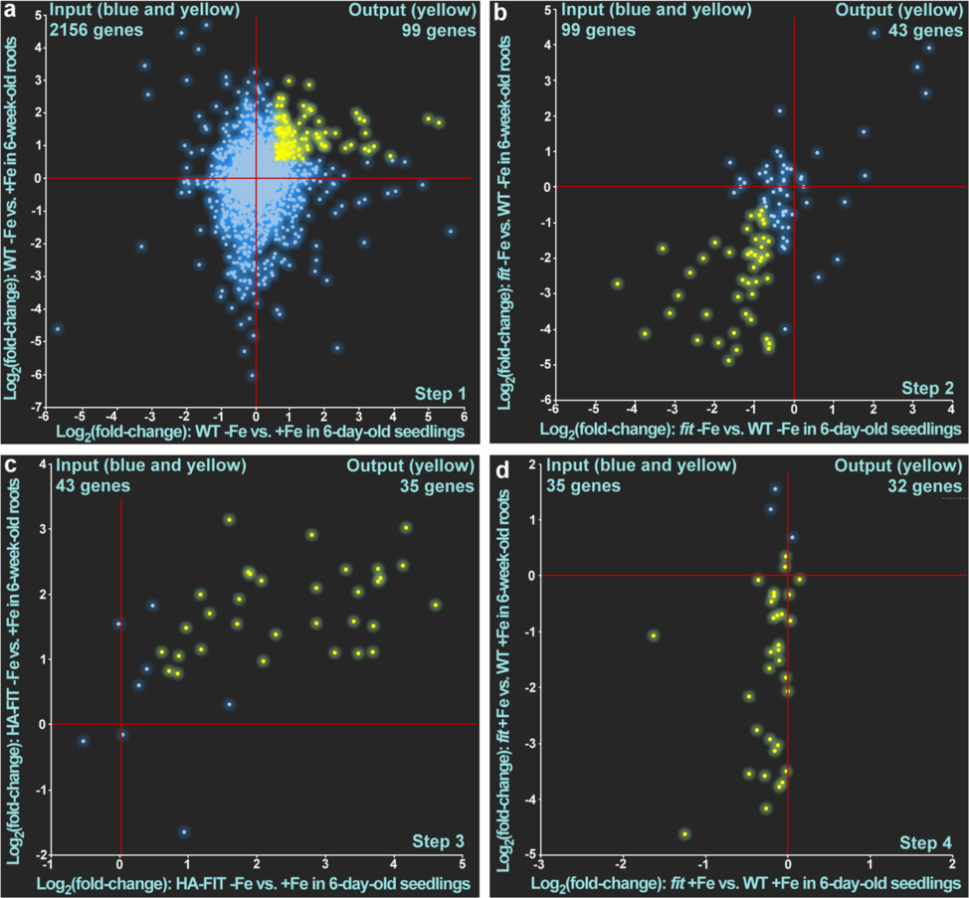
Four-step filtering of FIT-induced genes using scatterplot analysis of log_2_ fold changes of gene expression in the respective comparison in seedlings (horizontal) and roots (vertical). The blue dots represent genes that did not match the requirement and were removed in the subsequent step. The yellow dots represent gene expression patterns that matched the requirement and which were used as the input for the subsequent pattern analysis. The respective zero-points are indicated by red crosshairs. The genes filtered in **a** were used as input in **b**. The genes filtered in **b** were used as input in **c**. The genes filtered in **c** were used as input in **d**. The yellow dots in **d **represent the 32 FIT-induced genes (Tables 1 and 2)

Next, we performed a consecutive four-step filtering of genes by scatter plot analysis. In the first step we selected 99 genes that were induced by -Fe in the wild type in the comparisons WT -Fe vs. WT  +Fe in roots and seedlings (Fig. 3a, Additional file 5: Table S2). In the second step we selected genes that were down-regulated under iron deficiency in the *fit* knock-out mutant compared to wild type (Fig. 3b, Additional file 5: Table S2). In the third step we filtered out genes that were up-regulated in the comparison HA-FIT at -Fe vs. +Fe in roots and seedlings (Fig. 3c, Additional file 5: Table S2) since the FIT target genes *IRT1* and *FRO2* are only induced under iron deficient conditions in a constitutive FIT over-expressor [11, 12, 28]. In the last step, we selected genes that were not induced in *fit* vs. WT under  +Fe in roots and seedlings (Fig. 3d, Additional file 5: Table S2). As a result, we ended up with 32 genes that we considered as positively FIT-regulated (Tables 1 and 2, Additional file 5: Table S2). As these were found regulated by FIT in roots and seedlings, we refer to them as robustly FIT-induced. Out of these 32 genes, 21 have been related to FIT before [11, 12]. However, 11 genes (AT1G32380, AT1G14182, AT1G14185, AT2G35850, AT4G17680, AT1G09560, AT5G62420, AT5G45105, AT5G55250, AT1G53635 and AT5G46060) are novel FIT-induced genes previously not known in the FIT regulation context (Table 2). A comparison of the expression pattern analysis with the results of hierarchical clustering shows that all the genes found in the expression analysis were also present in at least one of the iron- and FIT-associated clusters (Additional file 5: Table S1).

**Table 1:**
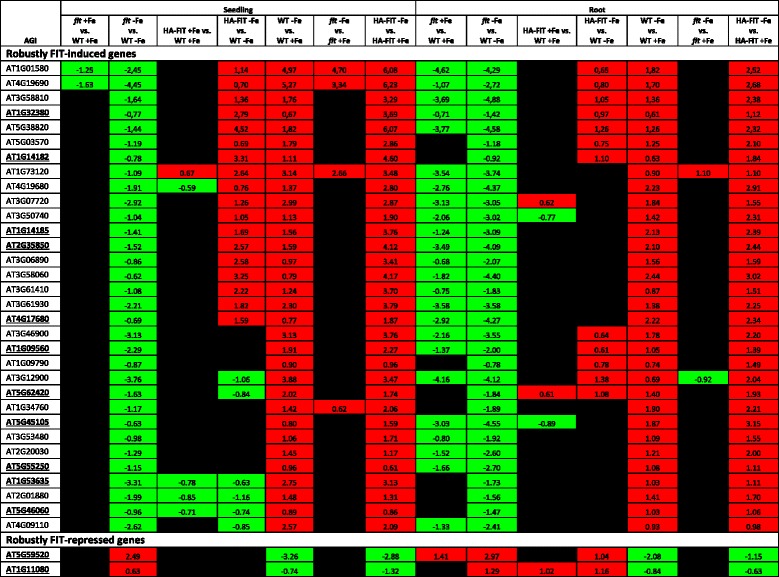
Expression patterns of the robustly FIT-regulated genes in six-day-old seedlings and six-week-old roots

**Fig. 4:**
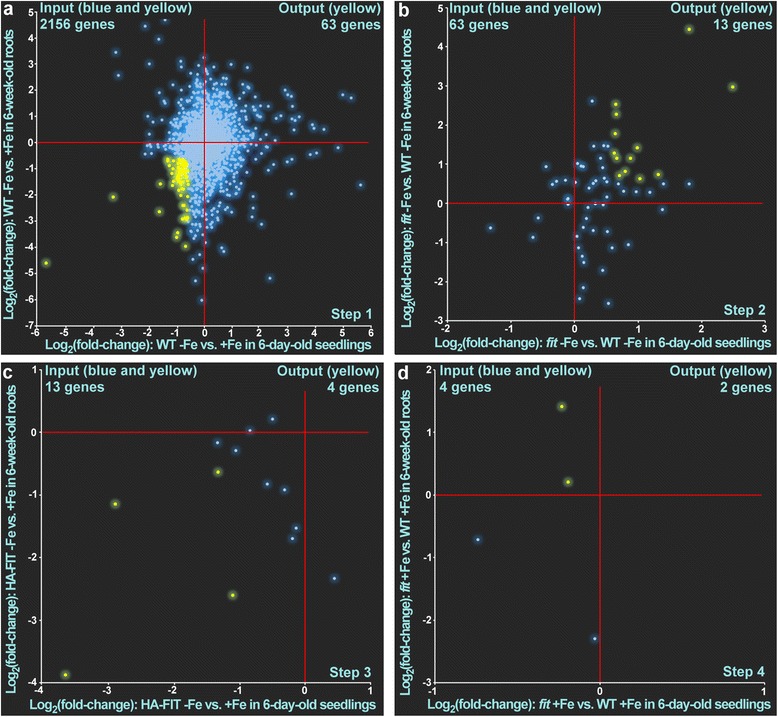
Four-step filtering of FIT-repressed genes using scatterplot analysis of log_2_ fold changes of gene expression in the respective comparison in seedlings (horizontal) and roots (vertical). The blue dots represent genes that did not match the requirement and were removed in the subsequent step. The yellow dots represent gene expression patterns that matched the requirement and which were used as the input for the subsequent pattern analysis. The respective zero-points are indicated by red crosshairs. The genes filtered in **a** were used as input in **b**. The genes filtered in **b** were used as input in **c**. The genes filtered in **c** were used as input in **d**. The yellow dots in **d** represent the FIT-repressed genes (Tables 1 and 2)

The genes were identified by expression pattern analysis. The selection criteria for FIT-induced genes were: induced under -Fe in WT, repressed in *fit* vs. WT at -Fe, induced in HA-FIT under -Fe and not induced in *fit* vs. WT at  +Fe (Fig. 3). Those for FIT-repressed genes were: repressed under -Fe in WT, induced in *fit* vs. WT at -Fe, repressed in HA-FIT under -Fe and not repressed in *fit* vs. WT at  +Fe (Fig. 4). These criteria had to be met in roots and seedlings. The given values are log_2_(fold change). Up-regulation is indicated by red background, down-regulation is indicated by green background and insignificant or below threshold regulation is indicated by black background. AGI codes of genes that have been previously associated with FIT [11] and the FIT-regulated gene *FRO2* [11, 12] are written normal, the AGI codes of the novel robustly FIT-induced genes are written in bold and underlined. For more information on the genes see Table 2

**Table 2:** Symbols or descriptions of the robustly FIT-regulated genes

AGI	Symbol or shortened Description
Robustly FIT-induced genes	
AT1G01580	FERRIC REDUCTION OXIDASE 2 (FRO2)
**AT1G09560**	GERMIN-LIKE PROTEIN 5 (GLP5)
AT1G09790	COBRA-LIKE PROTEIN 6 PRECURSOR (COBL6)
**AT1G14182**	SCR-LIKE 28 (SCRL28)
**AT1G14185**	Glucose-methanol-choline (GMC) oxidoreductase family protein
**AT1G32380**	PHOSPHORIBOSYL PYROPHOSPHATE (PRPP) SYNTHASE 2 (PRS2)
AT1G34760	GENERAL REGULATORY FACTOR 11 (GRF11)
**AT1G53635**	unknown protein
AT1G73120	unknown protein
AT2G01880	PURPLE ACID PHOSPHATASE 7 (PAP7)
AT2G20030	RING/U-box superfamily protein
**AT2G35850**	unknown protein
AT3G06890	unknown protein
AT3G07720	Galactose oxidase/kelch repeat superfamily protein
AT3G12900	2-oxoglutarate (2OG) and Fe(II)-dependent oxygenase superfamily protein
AT3G46900	COPPER TRANSPORTER 2 (COPT2)
AT3G50740	UDP-GLUCOSYL TRANSFERASE 72E1 (UGT72E1)
AT3G53480	ATP-BINDING CASSETTE G37 (ABCG37)
AT3G58060	Cation efflux family protein
AT3G58810	METAL TOLERANCE PROTEIN A2 (MTPA2)
AT3G61410	BEST Arabidopsis thaliana protein match is: U-box domain-containing protein kinase family protein (TAIR:AT2G45910.1)
AT3G61930	unknown protein
AT4G09110	RING/U-box superfamily protein
**AT4G17680**	SBP (S-ribonuclease binding protein) family protein
AT4G19680	IRON REGULATED TRANSPORTER 2 (IRT2)
AT4G19690	IRON-REGULATED TRANSPORTER 1 (IRT1)
AT5G03570	IRON REGULATED 2 (IREG2)
AT5G38820	Encodes a putative amino acid transporter
**AT5G45105**	ZINC TRANSPORTER 8 PRECURSOR (ZIP8)
**AT5G46060**	Protein of unknown function. DUF599
**AT5G55250**	IAA CARBOXYLMETHYLTRANSFERASE 1 (IAMT1)
**AT5G62420**	NAD(P)-linked oxidoreductase superfamily protein
Robustly FIT-repressed genes	
**AT5G59520**	ZRT/IRT-LIKE PROTEIN 2 (ZIP2)
**AT1G11080**	SERINE CARBOXYPEPTIDASE-LIKE 31 (scpl31)

If available the short symbols are given in brackets along with the fully written gene name. If no symbol was available we provided a shortened version of the description. The AGI codes of the novel robustly FIT-induced and repressed genes that had not been previously associated with FIT [11, 12] are written bold and underlined. For more information on the expression patterns of these genes see Table 1. The genes in this table were selected by their expression patterns in roots and seedlings

The question whether there are also FIT-repressed genes was addressed with the inverse analysis as above. In the first step we selected 63 genes that were repressed by -Fe in the wild type in the comparisons WT -Fe vs. WT  +Fe in roots and seedlings (Fig. 4a). In the second step we selected genes that were up-regulated under iron deficiency in the *fit* knock-out mutant compared to wild type (Fig. 4b). In the third step we filtered out genes that were down-regulated in the comparison HA-FIT at -Fe vs. +Fe in roots and seedlings (Fig. 4c). In the fourth step, we selected genes that were not repressed in the absence of FIT under  +Fe in the comparison *fit*  +Fe vs. WT  +Fe in roots and seedlings (Fig. 4d). As a result, we ended up with 2 genes that we considered as repressed by FIT (Tables 1 and 2). The two FIT-repressed genes were *SERINE CARBOXYPEPTIDASE-LIKE 31* (*SCPL31*, AT1G11080) and *ZRT/IRT-LIKE PROTEIN 2* (*ZIP2*, AT5G59520). SCPL proteins are annotated to have peptidase activity by sequence similarities but a number of SCPL, instead of peptidase activity, act as lyases and acyltransferases in the production of secondary metabolites involved in herbivory defense or UV protection [33]. However, the catalytic activity and the biological processes in which SCPL31 might be involved have not yet been determined. Excess zinc causes secondary iron deficiency in *A. thaliana* and iron uptake genes are induced to compensate for secondary iron deficiency [34]. Another zinc transporter, *ZIP8*, belongs to the robustly FIT-induced genes (Tables 1 and 2). It can be speculated that under iron deficiency and in situations of excess zinc, zinc homeostasis could be modulated by FIT to reduce the negative effects of zinc on iron homeostasis.

### Expression pattern analysis in six-day-old seedlings and six-week-old roots reveals distinct sets of FIT-dependent genes

**Fig. 5:**
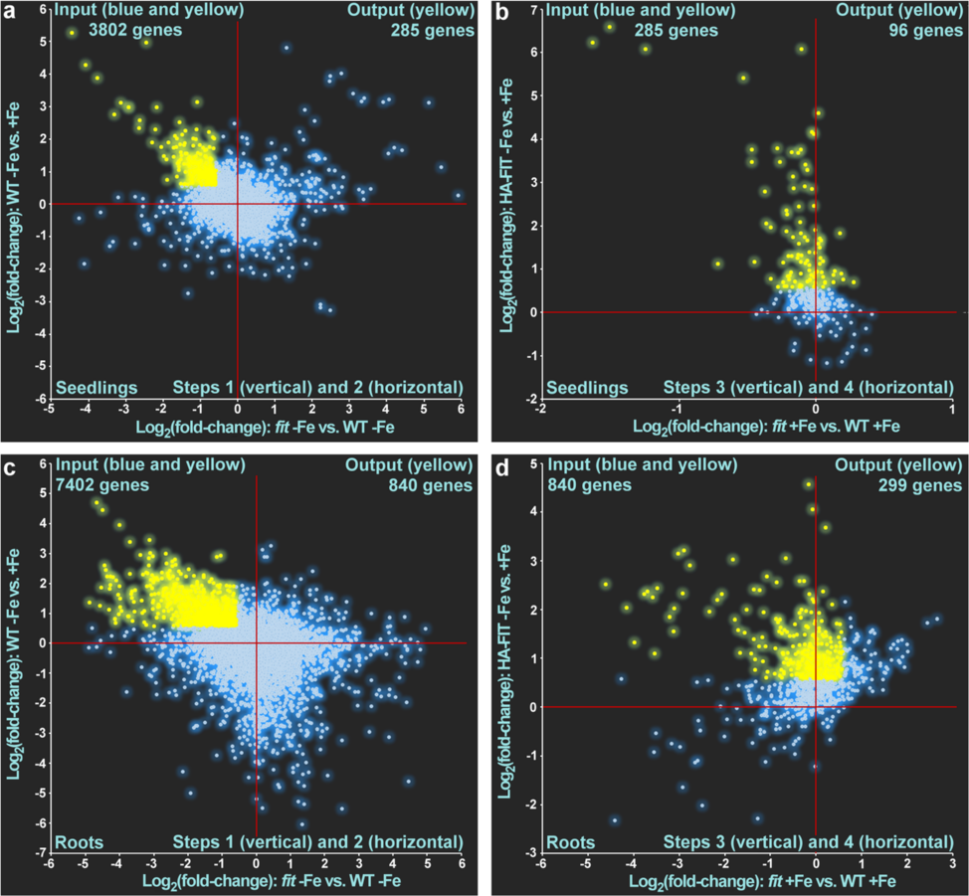
Filtering of temporally FIT-induced genes using scatterplot analysis of log_2_ fold changes of gene expression in the respective comparisons in seedlings (**a** and **b**) and roots (**c** and **d**). The blue dots represent genes that did not match the requirement and were removed in the subsequent step. The yellow dots represent gene expression patterns that matched the requirements and which were used as the input for the subsequent pattern analysis. The respective zero-points are indicated by red crosshairs. The genes filtered in **a** were used as input in **b**. The genes filtered in **c** were used as input in **d**. The yellow dots in **b** and **d** represent FIT-induced genes in six-day-old seedlings (**b**) (Additional file 5: Table S3) and in roots of sic-week-old plants (**d**) (Additional file 5: Table S4). The filtering steps 1 and 2 as well as 3 and 4 are combined in one graph, respectively

We used the same filtering as above to detect genes regulated in a FIT-dependent manner only in six-day-old seedlings or only in six-week-old roots, respectively. In seedlings, out of the 3802 input genes (red circle in Fig. 1m, Additional file 3: Dataset 2) that were found regulated in one of the comparisons, 285 were expressed at a higher level in WT -Fe vs. +Fe and expressed at a lower level in *fit* -Fe vs. WT -Fe (Fig. 5a). Out of these 285 genes, 96 were expressed at a higher level in the comparison HA-FIT -Fe vs. HA-FIT  +Fe and not expressed at a higher level in the comparison *fit* -Fe vs. *fit*  +Fe (Fig. 5b). Among these 96 genes there were all the 32 previously found FIT-regulated genes, as expected. Hence, 64 genes were regulated in a FIT-dependent manner exclusively in six-day-old seedlings (Additional file 5: Table S3).

In roots of six-week-old plants, out of 7402 total regulated genes (blue circle in Fig. 1m, Additional file 3: Dataset 1) 840 were expressed at a higher level in WT -Fe vs. +Fe and expressed at a lower level in *fit* -Fe vs. WT -Fe (Fig. 5c). Out of these 840 genes 299 were expressed at a higher level in HA-FIT -Fe vs. HA-FIT  +Fe and not expressed at a higher level in *fit* -Fe vs. *fit*  +Fe (Fig. 5d). Also these genes comprised the 32 previously determined FIT-regulated genes, so that finally, 267 genes were found regulated in a FIT-dependent manner exclusively in six-week-old roots (Additional file 5: Table S4).

Among the 64 genes that were regulated in a FIT-dependent manner specifically in seedlings there were 15 genes that have been previously associated with FIT [11] (Additional file 5: Table S3) and those that were regulated in a FIT-dependent manner specifically in six-week-old roots comprised 9 previously FIT-associated genes (Additional file 5: Table S3). Thus, including the above-described 32 robustly FIT-induced genes, our results cover 45 out of the 72 previously known FIT-associated genes [11]. Three hundred eighteen FIT-dependent genes were not previously described as FIT-regulated genes. Eleven of them were stably regulated in a FIT-dependent manner in six-day-old seedlings and in six-week-old roots. Forty-nine were regulated in a FIT-dependent manner exclusively in six-day-old seedlings. Two hundred fifty-eight genes displayed a FIT-dependent regulation pattern exclusively in six-week-old roots. Interestingly, 11 of the seedling-specific FIT-dependent genes were not found regulated at all in six-week-old roots. These are considered generally seedling-specific. One hundred sixty-five of the FIT-dependent genes in six-week-old roots were not found regulated at all in seedlings. These are considered generally root-specific. The generally root-specific genes contained four previously known FIT targets which coincides with the fact that previously found FIT targets were obtained with roots of plants in the four to six true leaf stage plus three days of treatment [11]. The 64 seedling-specific, positively FIT-dependent genes comprise a total of 11 genes that were either previously determined as FIT targets [11] or that were shown to play specific roles in iron homeostasis [4, 35–39] (see also Additional file 5: Table S3 column S). Additionally, these genes contain NADK1 (AT3G21070), an NAD kinase which is involved in de novo synthesis of NADP [40]. As suggested before [41, 42], reducing equivalents are required to maintain the iron uptake machinery and de novo synthesis of NADP could be increased as a response to these requirements.

**Fig. 6:**
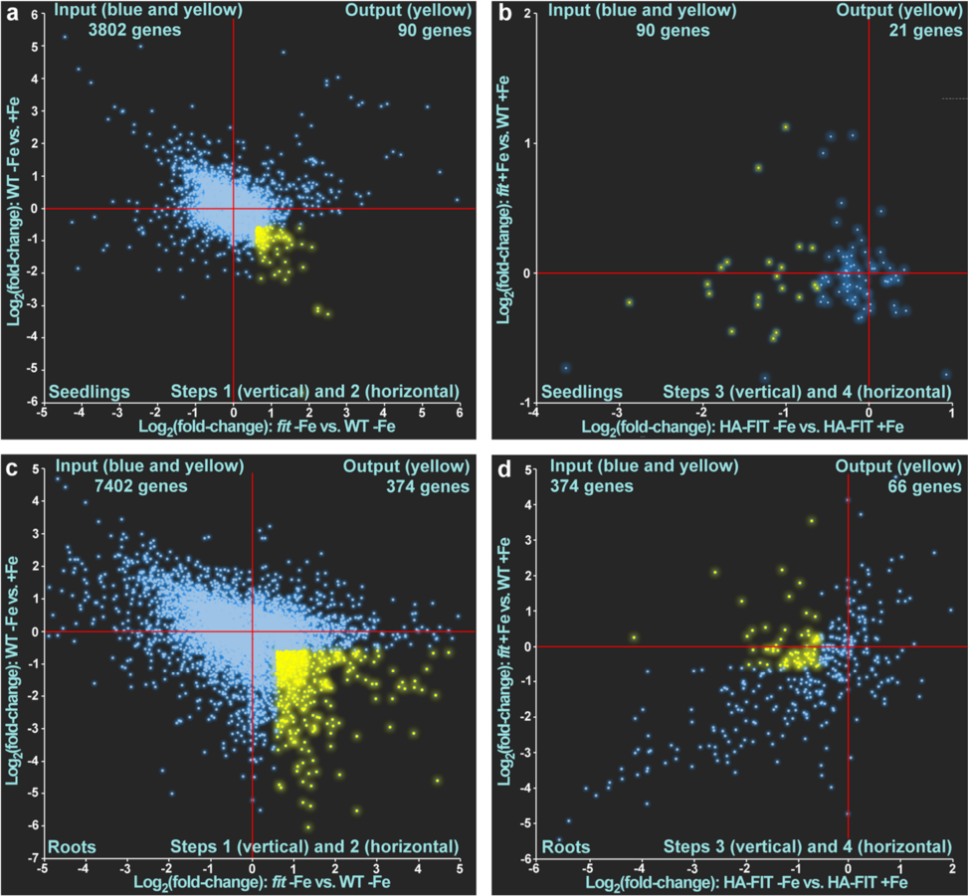
Filtering of temporally FIT-repressed genes using scatterplot analysis of log_2_ fold changes of gene expression in the respective comparisons in seedlings (**a** and **b**) and roots (**c** and **d**). The blue dots represent genes that did not match the requirement and were removed in the subsequent step. The yellow dots represent gene expression patterns that matched the requirements and which were used as the input for the subsequent pattern analysis. The respective zero-points are indicated by red crosshairs. The genes filtered in **a** were used as input in **b**. The genes filtered in **c** were used as input in **d**. The yellow dots in **b** and **d** represent FIT-repressed genes in six-day-old seedlings (**b**) (Additional file 5: Table S5) and in roots of sic-week-old plants (**d**) (Additional file 5: Table S6). The filtering steps 1 and 2 as well as 3 and 4 are combined in one graph, respectively

We also performed the same analysis with inversed parameters to find genes that were repressed in a FIT-dependent manner in either seedlings or root samples (Fig. 6). After subtraction of the above-determined two robustly FIT-repressed genes, another 64 genes were repressed by FIT in six-week-old roots (Additional file 5: Table S5) and 19 genes in six-day-old seedlings (Additional file 5: Table S6). Taken together, we suggest as a possible explanation that along with the robustly FIT-dependent genes other distinct sets of genes could be under the control of additional but yet unknown factors which might act in different developmental stages.

We suspected that the genes which showed the FIT-dependent regulation pattern only in the seedling samples but not in the root samples (in total 83 genes designated as FIT-repressed/induced only in six-day-old seedlings, Fig. 8) were expressed in roots where FIT is active. Sixty-six of these 83 genes were found in our study to be FIT-regulated in roots. The other 17 genes were checked for root expression using publicly available microarray and RNA-seq data via the Genevestigator tool [43]. All 11 FIT-induced and four FIT-repressed genes out of these 17 genes were indeed all found expressed at low, medium or high level in root and root cell samples. Only two FIT-repressed genes (AT1G67265 and AT4G38825) were found expressed at very low level in roots and were therefore excluded from any further analyses. We cannot exclude that some other genes which might have been expressed at a very low level in roots but higher level in cotyledons were not detected in our analyses.

The robustly FIT-regulated genes comprise a number of transporters that are involved in iron uptake, such as IRT1 [7, 44], or in sequestration of other bivalent metals under iron deficiency such as MTPA2 [45], MTP8 [46] and IREG2 [47]. COPT2 is involved in copper uptake [22]. *COPT2* expression and copper uptake are increased under Fe deficiency, possibly to supply Cu to enzymes that use Cu as a cofactor [22]. The exact function of the ZRT/IRT-like family protein ZIP8 is unknown but it could potentially be an Fe or Zn transporter. AT5G38820 is a putative amino acid transporter. The FIT-repressed gene *ZIP2* encodes a transporter that is localized to the plasmamembrane and capable of transporting Zn and Mn [48]. The role of ZIP2 in iron homeostasis is unclear but it might also be involved in Zn or Mn detoxification. IRT2 is an iron transporter. *IRT2* expression is induced by iron and zinc deficiency [49, 50]. PDR9 might be an exporter of scopoletin and derivates into the rhizosphere [51].

Some robustly FIT-regulated genes encode enzymes. FRO2 is a ferric chelate reductase that is part of the iron uptake machinery in Arabidopsis [6]. PAP7 is a purple acid phosphatase that is targeted to peroxisomes [52]. Peroxisomes are involved in a number of metabolic pathways but also in the response to oxidative stress, JA and SA biosynthesis and indole-3-butyric acid metabolism [53]. Hence, PAP7 could play a role in the regulation of such processes under Fe deficiency through reversible protein phosphorylation [53]. *PAP7* regulation also depends on JAI3. Thus, in addition to FIT, it may be regulated by MYC2 [54]. AT1G14185 is a glucose-methanol-choline (GMC) oxidoreductase family protein with unclear function. PRS2 is a phosphoribosyl pyrophosphate synthetase. According to BioCYC [55] the product, 5-phospho-α-D-ribose 1-diphosphate, could serve as a precursor in several nucleoside and nucleotide salvage pathways but could also be a precursor of NAD^+^ which might be required in higher amounts under iron deficiency. IAMT1 converts IAA to methyl-IAA (MeIAA). MeIAA is an inactive form of IAA that gets converted back into IAA by hydrolysis [56]. It has been suggested that the nonpolar and mobile MeIAA molecule serves to quickly change local IAA concentrations [56]. UGT72E1 is involved in the glycosylation of sinapyl aldehyde and coniferyl aldehyde [57]. The phenylpropanoid glucosides are better soluble than their non-glycosylated forms and ready for transport. Coniferyl aldehyde and sinapyl aldehyde can be precursors of ferulic acid, sinapic acid and lignin. It has been suggested that glycosylation of these phenylpropanoids might regulate the biosynthesis of lignin and the metabolism of a number of other phenylpropanoids [57]. AT5G62420 is an NAD(P)-linked oxidoreductase superfamily protein of unknown function. COBL6 is predicted to be anchored to the plasmamembrane [58] and has been previously annotated as a putative phytochelatin synthase [11, 47]. The FIT-regulated genes also encompass the genes of three putative E3 ligases: AT2G20030 and AT4G09110 are RING/U-box superfamily proteins and AT3G61410 contains a U-box. An enzymatic function of these gene products has not been demonstrated but based on the similarity to E3 ligases we speculate that they might be involved in the regulation of proteasome-dependent protein turnover under iron deficiency. AT3G07720 is a galactose oxidase/kelch repeat superfamily protein with high similarity to nitrile specifier proteins which are involved in glucosinolate breakdown [59]. AT3G12900 shows a high similarity to AT3G13600 (F6’H1). Therefore, we speculate that it could also play a role in coumarin biosynthesis or metabolism. *SCPL31* which is the other FIT-repressed gene, encodes a putative serine carboxypeptidase. Enzymatic activity has not been demonstrated but the protein could play a role in proteasome-independent protein processing or turnover.

The exact molecular function of another fraction of the robustly FIT-induced genes is unknown. The 14-3-3 protein GRF11 has been demonstrated to act downstream of NO and has been suggested to modulate FIT expression in a feedback loop [60]. GLP5 is a plasmodesmata-located protein. *GLP5* over-expressing plants display reduced primary root and enhanced lateral root growth [61]. Hence, GLP5 might be involved in altering the root architecture under iron deficiency. AT4G17680 is an SBP (S-ribonuclease binding protein) family protein it contains a Zinc finger domain. We speculate that this might be a regulatory protein, possibly by taking a role in mRNA processing. SCRL28 is a 97 amino acids long peptide and member of a family of small, secreted, cysteine rich proteins. According to UniProtKB [62] it is a putative defensin-like protein. The role of SCRL28 is unknown. *AT1G73120*, the gene of an unknown protein has been demonstrated to be induced under excess Zn [34]. The genes of five more unknown proteins are among the robustly FIT-regulated proteins: *AT3G06890*, *AT3G61930*, *AT1G53635*, *AT2G35850* and *AT5G46060. AT3G06890*, *AT3G61930*, *AT1G53635* and *AT2G35850* encode 79 to 128 amino acids long peptides that might have regulatory functions or play roles in signal transduction.

### Validation of FIT-dependent genes by assembly of a virtual dataset

We assembled a virtual dataset using our own expression data of iron-regulated genes together with the transcriptomic data from 9 previous studies in which Arabidopsis wild type roots or seedlings were tested for transcriptomic adaptations upon iron deficiency [11, 13, 19, 20, 24, 25, 63–65]. From time course experiments we used the 24 h [24], 48 h and 72 h data [64]. The reconstructed data from two publications [11, 20] were incomplete since they only contained induced genes. Together, 14 transcriptomic analyses from 9 studies and our own data have been taken into account (Additional file 3: Dataset 5). From the collected data we assembled a virtual iron regulation dataset (Additional file 3: Dataset 6) in which the genes were filtered by the number of occurrences among the regulated genes and by the uniformity of their regulation. From 5851 genes that were found regulated in at least one of the studies, 598 genes met the requirements of which 437 genes were induced and 161 were repressed under -Fe. Out of the 32 FIT-regulated genes all but seven genes (AT1G14182, AT1G32380, AT1G53635, AT4G17680 AT5G4510, AT5G46060 and AT5G55250) were among the induced genes in this virtual dataset. A closer look at these seven genes showed that five of them (AT1G14182, AT1G53635, AT4G17680, AT5G45105 and AT5G46060) had not been included in the Affymetrix ATH1 chips used in the published work. The two other genes, AT1G32380 and AT5G55250, were barely 1.5 and 2-fold up-regulated under -Fe, which makes them prone to be filtered by the oftentimes used two-fold detection threshold.

One observation that we made during the assembly of the virtual dataset was the very variable number of genes detected as regulated in the distinct analyses. The highest number of genes that was found regulated in wild type upon iron deficiency was 2673 (this study). A comparable number of genes was for example also detected by Long et al. [25] in the 48 h and 72 h time points of the time course analysis. The lowest number of genes that were found regulated upon iron deficiency in wild type roots was 14 [65] while in transcriptomic analyses of other studies this number ranged from roughly 150 to 1000 genes. The average number is ca. 800. Hence, it is not surprising that potentially important genes were often not detected and this might have contributed to the fact that AT5G55250 did not make it into the virtual dataset and that some of the newly FIT-associated genes were not found as such. A reason for the great variability of the number of detected differentially expressed genes could be inconsistent growth conditions which may lead to a high variance between the biological replicates and consequently to insignificant regulation. Interestingly, even the central regulator of iron uptake, FIT, has only been found regulated in 7 of 14 analyses. This might be due to the fact that FIT is relatively weakly up-regulated under iron deficiency but might also be due to the fact that this gene is only present on two of four often used microarrays to this time point. Hence, detection of some important genes might also depend on the microarrays used to perform the transcriptomic analysis. The comparison of the 598 genes in the virtual dataset with our own data showed that we found 293 of these genes regulated while 305 genes in the virtual dataset were not found regulated in our analyses. The fact that the average VIRT absolute value was 0.36 shows that our analyses with ca. 49 % covered an above-average number of these genes.

### Robust marker genes for iron deficiency

Our virtual dataset of the transcriptomic response of wild type to iron deficiency was constructed so that the genes were not only ranked by the number of occurrences in all analyses but also according to the uniformity of their regulation across multiple analyses. Setting thresholds in the process of constructing the dataset enabled us to filter the regulatory noise and pinpoint those genes that are most reliably induced upon iron deficiency in wild type Arabidopsis roots and whole seedlings. According to this procedure, 598 out of 5847 genes that were found regulated in at least one of the 14 analyses of WT -Fe vs. WT  +Fe in this study and all the other considered studies [11, 13, 19, 20, 24, 25, 63–65] are present in the virtual dataset (Additional file 3: Dataset 6). This number is ca. 25 % less than the average number of genes that were found regulated in all considered studies. The highest ranked up- and down-regulated genes are shown in Table 3. The four topmost ranked induced genes upon iron deficiency in WT are AT3G07720 (galactose oxidase, kelch repeat family protein), AT3G58810 (*MTPA2*), AT4G19690 (*IRT1*), AT3G12900 (2-oxoglutarate (2OG) and Fe(II)-dependent oxygenase superfamily protein) and AT3G61930 (unknown protein) (Table 3). This makes them the most reliable marker genes for iron deficiency in Arabidopsis roots and seedlings. AT3G07720 and MTPA2 match previous findings [13]. AT4G19690, AT3G12900 and AT3G61930 are almost equivalent alternatives albeit their ranking is slightly lower. Although stably up-regulated under iron deficiency in roots and seedlings, not much is known about the functions AT3G12900 and AT3G61930. Due to its similarity to AT3G13610 (F6'H1) it can be guessed that AT3G12900 possibly also participates in coumarin biosynthesis. The consistent induction of these genes upon iron deficiency, along with the hitherto unknown AT3G07720, they are interesting new targets for future research. Interestingly, among the top 12 up-regulated genes 11 have been associated with FIT by Colangelo and Guerinot [11] and this study. Only AT3G56980 (*bHLH039*) is regulated independently from FIT. This fits the previous finding that *bHLH039* is regulated together with *bHlh038*, *bHLH100* and *bHLH101* by the concerted action of bHLH104 and ILR3 [66].

**Table 3:** Genes that were found most stably up or down-regulated in Arabidopsis wild type across 11 studies in a total of 14 transcriptomic comparisons between -Fe and  +Fe

Symbol	AGI	1	2	3	4	5	6a	6b	6c	7	8	9a	9b	10	11	VIRT
Genes that are most stably up-regulated under iron deficiency																
Kelch repeat family protein	AT3G07720	1	1	1	1	1	1	1	1	1	1	1	1	1	1	1.00
MTPA2	AT3G58810	1	1	1	1	1	1	1	1	1	1	1	1	1	1	1.00
IRT1	AT4G19690	1	1	1	1	1	1	1	1	1	1	1	1	1	0	0.93
2OG	AT3G12900	1	1	1	1	1	1	1	1	1	1	1	1	1	0	0.93
unknown	AT3G61930	1	1	1	1	1	1	1	1	1	1	1	1	1	0	0.93
GLP5	AT1G09560	1	1	1	1	1	1	1	1	1	1	1	1	0	0	0.86
Unknown	AT3G06890	1	1	1	1	1	1	1	1	0	1	1	1	1	0	0.86
COPT2	AT3G46900	1	1	0	1	1	1	1	1	1	1	1	1	1	0	0.86
UGT72E1	AT3G50740	1	1	1	1	1	1	1	1	0	0	1	1	1	1	0.86
bHLH039	AT3G56980	1	1	1	1	1	1	1	1	0	1	1	1	0	1	0.86
MYB72	AT1G56160	1	1	0	1	1	1	1	1	1	1	1	1	1	0	0.86
Cation efflux family protein	AT3G58060	1	1	1	1	1	1	1	1	0	1	1	1	1	0	0.86
Genes that are most stably down-regulated under iron deficiency																
FER1	AT5G01600	-1	0	-1	-1	-1	-1	-1	-1		-1	-1	-1		0	-0.83
ATABC1	AT4G04770	-1	0	-1	-1	-1	-1	-1	-1		0	-1	-1		0	-0.75
unknown	AT2G36885	-1	0	0	0	-1	-1	-1	-1		-1	-1	-1		0	-0.67
PSAF	AT1G31330	-1	0	-1	-1	0	-1	-1	0		0	-1	-1		0	-0.58
uncharacterized protein family (UPF0016)	AT1G68650	-1	0	0	0	-1	-1	-1	-1		-1	0	-1		0	-0.58
PER21	AT2G37130	-1	0	-1	-1	0	-1	-1	0		0	-1	-1		0	-0.58
FER4	AT2G40300	-1	0	0	-1	-1	-1	-1	-1		-1	0	0		0	-0.58
LAC7	AT3G09220	-1	-1	0	0	-1	-1	-1	0		0	-1	-1		0	-0.58
SAPX	AT4G08390	-1	0	-1	-1	0	-1	-1	0		0	-1	-1		0	-0.58
unknown	AT5G59400	-1	0	-1	-1	-1	-1	-1	-1		0	0	0		0	-0.58
HEMA1	AT1G58290	0	0	0	0	-1	-1	-1	-1		0	-1	-1		0	-0.50
FSD1	AT4G25100	-1	-1	0	-1	-1	0	0	-1		0	0	0		-1	-0.50
peroxidase, putative	AT5G64100	0	-1	0	-1	0	-1	-1	0		0	-1	-1		0	-0.50

Up-regulated genes are represented by the value 1. Down-regulated genes are represented by the value -1. Genes with no significant regulation or with regulation below the threshold of the respective study are represented by the value 0. The genes with the highest VIRT absolute value are regarded as most stably up- or down-regulated, respectively. 1: This study (seedlings). 2: This study (roots). 3: Bauer and Blondet, 2011 [63]. 4: Ivanov et al., 2012 [13]. 5: Yang et al., 2010 [19]. 6a: Long et al., 2010. 48 h [25]. 6b: Long et al., 2010. 72 h [25]. 6c: Long et al., 2010. 24 h (WT vs. *pye*) [25]. 7: Garcia et al., 2010 [20]. 8: Buckhout et al., 2009 [24]. 9a: Dinneny et al., 2008. 48 h [64]. 9b: Dinneny et al., 2008. 72 h [64]. 10: Colangelo and Guerinot, 2004 [11]. 11: Schuler et al., 2011 [65]. For the down-regulated genes, the analyses 7 [20] and 10 [11] were excluded in the calculation of the VIRT value since these only contained genes that were induced upon iron deficiency

Among the most stably up-regulated genes under iron deficiency there is also the newly FIT-associated gene AT1G09560 (*GLP5*). GLP5 is a germin-like protein. Germin-like proteins have been associated with pathogen response [67]. It is possible that germin-like proteins also play a role in other stress responses such as iron deficiency. GLP5, also named PGLP1, is a component of the NCAP (non-cell-autonomous protein) pathway, locates to plasmodesmata and regulates root growth [61]. So GLP5 could be involved in iron signal complex translocation or in the altered root growth as a response to iron deficiency. Hence it would be interesting to know whether adaptations of the root architecture to iron deficiency is disturbed by *glp5* knock-out or *GLP5* over-expression mutants.

The three topmost ranked down-regulated genes are AT5G01600 (*FER1*), AT4G04770 (*ATABC1*) and AT2G36885 (unknown protein) (Table 3). However, since their rank absolute value is lower than the five topmost induced genes they are less suitable as robust iron deficiency marker genes. However, due to its ranking the down-regulated gene that is best-suited for this purpose would be *FER1*.

### Co-expression and functional analysis of the virtual dataset revealed functionally enriched regulons

**Fig. 7:**
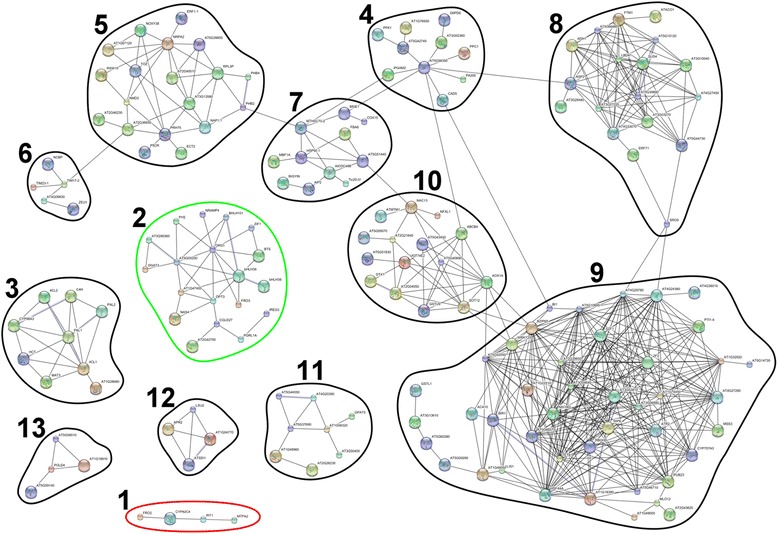
Co-expression network built from the genes induced under -Fe in the virtual dataset: Regulon 1: contains members of the FIT target network [13]. Regulon 2: consists of members of the iron homeostasis network [13]. Regulon 3: is largely composed of genes involved in phenylpropanoid metabolism. Regulon 4: mainly comprises genes that participate in the pentose phosphate pathway, glycolysis and gluconeogenesis. Regulon 5: is mostly composed of genes that are involved in RNA processing and translation. Regulon 6: contains mitochondrial proteins. Regulon 7: is heterogeneous but contains comparably many chaperons. Regulon 8: is enriched in genes involved in amino acid metabolism. Regulon 9: is also heterogeneous but enriched in genes that participate in plant-pathogen interaction. Regulon 10: shows no enrichment of molecular functions. Regulon 11: mainly contains genes that participate in purine, lipid and aromatic compound metabolism. Regulon 12: is composed of genes involved in the response to low sulfur. Regulon 13 shows no enrichment of molecular functions. The network has been created with the String version 10 protein interaction database [27]. The confidence was set to ‘medium’ (0.400) and no genes were added. The 437 genes induced under -Fe in the virtual dataset were used as input. Singlet nodes have been removed and only networks with 4 or more nodes are shown. The resulting network image contains 169 genes (Additional file 5: Table S7). For a high resolution image see Additional file 6: Figure S4

Out of the 598 genes in the virtual dataset 437 were found induced. This is concordant with the general observation that under iron deficiency more genes are induced than repressed. We used these genes to create co-expression networks using the String version 10 tool [27]. Genes resulting in singlet nodes and networks with ≤ 3 nodes were disregarded. One hundred sixty-nine genes grouped into networks with ≥4 nodes (Fig. 7, Additional file 6: Figure S4). After rearranging the nodes we could detect a total of 13 networks with 4 to 43 nodes. Six networks (Fig. 7, regulons 1, 2, 3, 11, 12 and 13) had no connection to other networks. Seven networks (Fig. 7, regulons 4-10) were connected with each other by sharing few nodes.

One closed regulon (Fig. 7, regulon 1) contained genes of the FIT target network [13], namely *IRT1*, *MTPA2*, *FRO2* and *CYP82C4* (Additional file 5: Table S7). Among the input genes of the virtual dataset further members of this regulon (AT1G34760, AT1G73120, AT1G74770, AT3G07720, AT3G12820, AT3G12900, AT3G50740, AT3G58810, AT4G19680, AT4G19690, AT4G30120, AT4G31940 and AT5G38820) were present. Another closed regulon (Fig. 7, regulon 2) was mainly composed of known members of the iron homeostasis PYE-BTS regulon [13]. Only 3 genes of the original regulon were missing, namely *PP2-A9*, the gene of an unknown protein (AT2G30760) and *IPT3*. Hence, this regulon is very consistently and almost entirely induced upon iron deficiency. Interestingly, this regulon contained 5 additional members compared to the original network, namely *IREG3*, *BHLH38*, *DJC77*, *PGR5-LIKE A* and *CGLD27*. Thus, the PYE-BTS regulon could be extended by 5 members. The third closed regulon (Fig. 7, regulon 3) was mainly composed of genes that are involved in phenylpropanoid metabolism. Increase of phenylpropanoid biosynthesis has been previously observed at the proteomic level [42]. Among others, *PAL1*, *PAL2*, *4CL1* and *4CL2*, which catalyze the very first steps in the phenylpropanoid pathway, were members of this regulon (Additional file 5: Table S7) and the respective proteins were also found induced upon iron deficiency. This network did not contain genes of enzymes that synthesize the final conversions. However, *F6’H1* and another gene that has been hypothesized to also participate in coumarin biosynthesis (AT3G12900) as well as the gene of the ABC transporter PDR9 (AT3G53480) which could be responsible for coumarin secretion into the rhizosphere were among the consistently iron deficiency-induced genes (Additional file 3: Dataset 6) but not directly connected to one of the regulons in our graph (Fig. 7). Furthermore, this regulon also contained *MAT3* which provides S-adenosylmethionine that, among others, serves as a methyl group donor in coumarin biosynthesis. Among others, organic acids such as malic acid were discussed to attract soil bacteria which might contribute to enhance iron uptake [68, 69]. Coumarins are excreted under iron deficiency [39, 51, 70, 71] and coumarins also play roles under other abiotic stresses such as osmotic stress [72]. Coumarins like Scopoletin also function as phytoalexins [73]. Besides mobilization of rhizospheric iron they could also serve to alter the rhizobiome.

At the proteomic level induction of glucose metabolism upon iron deficiency has been observed [41, 42]. We identified a regulon with 10 nodes (Fig. 7, regulon 4) that is induced under iron-deficiency. Eight of the ten nodes are genes which are involved in glucose metabolism, namely *G6PD6*, *IPGAM2*, *PEPC1*, *PFK1,* the gene of a 6-phosphogluconate dehydrogenase family protein (AT3G02360), the gene of a sugar isomerase family protein (AT5G42740), the gene of a phosphofructokinase family protein (AT1G76550) and the gene of a pyruvate kinase family protein (AT5G56350). Additionally, *FBA6* was found induced in a neighboring and connected regulon (Fig. 7, network 7). This clearly indicates an increase of glycolysis and the pentose phosphate pathway. Both were suggested to provide energy equivalents, organic acids and reducing equivalents [41].

Another network with 19 members (Fig. 7, regulon 5) contained mainly transcription and translation-related genes. Among those are *MDN1*, *NRPA2, NAP1;1, RPL3P*, *PRH75*, *ERF1-1*, the gene of a ribosomal protein L10 family protein (AT2G40010), the gene of the ribosomal protein S4 (AT5G39850), the gene of a ribosomal protein L30/L7 family protein (AT3G13580), the gene of a nonsense-mediated mRNA decay NMD3 family protein (AT2G03820) and the gene of a zinc finger (C2H2 type) family protein (AT2G36930) (Additional file 5: Table S7).

Noticeable enrichment in genes that are involved in the response to low sulfur were found in a small four-member network (Fig. 7, regulon 12): the tetratricopeptide repeat (TPR)-like superfamily protein gene *SDI1* (AT5G48850), *LSU2* (AT5G24660), *APR2* (AT1G62180) and the gene of another so far uncharacterized tetratricopeptide repeat (TPR)-like superfamily protein (AT1G04770). *SDI1* is regulated by FIT specifically in seedlings (this study). The largest regulon contained 48 tightly interwoven nodes (Fig. 7, regulon 9). A portion of the genes in this regulon is involved in the response to various chemical, biotic and abiotic stimuli such as response to chitin, response to water deprivation or response to other organism as well as response to stress. This regulon also contained the formerly known and partially newly determined FIT downstream targets *PUB23*, *F6’H1*, *GSTL1*, *RBOHD*, the gene of two unknown proteins (AT1G49000 and AT4G29780) and the gene of a glycine rich protein (AT3G04640) (Additional file 5: Table S7).

Twenty-five of the FIT target genes were contained in the virtual dataset and used as input genes. Only three of them were present in the resulting co-expression network. Since we intentionally did not add any genes, the respective bridging nodes were missing so they appeared as singlet nodes or networks with <4 nodes and were removed. For the same reason the FIT target network and the iron homeostasis PYE-BTS-regulon were not connected. However, the deeper analysis of the virtual dataset showed a comparably high concordance with previous observations at the proteomic level. Although the actual overlap between distinct genes and proteins in the transcriptomic and proteomic analyses is comparably low and single comparisons between the proteomic and transcriptomic regulation under iron deficiency showed a pronounced discrepancy between gene and protein regulation, the general adaptations of some metabolic and regulatory pathways that were observed at the protein level [41, 42] are mirrored at the transcriptome level.

## Conclusions

**Fig. 8:**
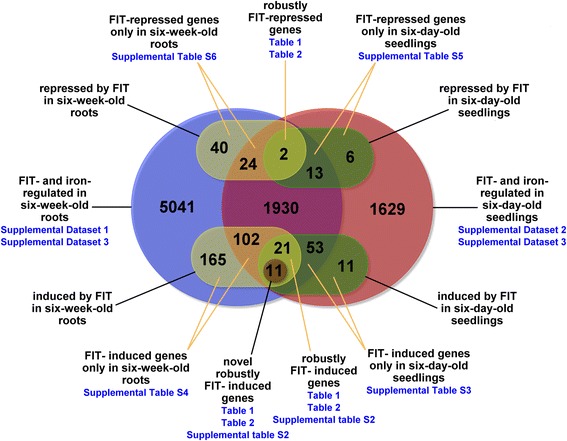
Summary of the results of our microarray analyses. The big blue circle represents genes that were found regulated in at least one comparison in six-week-old roots and the big red circle contains genes that were found regulated in at least one comparison in six-day-old seedlings. The lower yellow oval consists of genes that were found FIT-induced in six-week-old roots and the lower green oval represents the FIT-induced genes in six-day-old seedlings. The intersection between the lower yellow and green ovals contains the 32 genes that we consider robustly FIT-induced. Eleven of them are novel FIT-regulated genes (brown circle). We also detected FIT-repressed genes. The upper yellow oval represents genes that were found FIT-repressed in six-week-old roots and the upper green oval contains the FIT-repressed genes in six-day-old seedlings. The intersection between the upper yellow and green ovals contains the 2 genes that we consider robustly FIT-repressed

FIT is the central regulator of iron homeostasis in Arabidopsis. Until now, 73 genes were known to be regulated downstream of FIT [11, 12]. With stringent expression pattern analysis we divided the regulated genes in multiple subgroups with distinct expression patterns (Fig. 8). We were able to define 32 robustly FIT-induced genes among which there were 11 novel robustly FIT-induced genes. Additionally, we pinpointed two robustly FIT-repressed genes. Hence, for the first time a repressing effect of FIT could be demonstrated. Furthermore, our results indicate a total of 414 genes that were regulated in a FIT-dependent manner either in seedlings or in six-week-old roots. FIT influenced the expression of far more genes than previously demonstrated. We were able to show that the control by FIT also depends on hitherto unknown factors.

The construction of a virtual dataset based on 14 distinct transcriptomic analyses allowed for removing a great portion of regulatory noise and revealed a total of 598 genes that are stably regulated under iron deficiency in Arabidopsis roots and seedlings. Four hundred thirty-seven of them were found stably induced and 161 stably repressed under iron deficiency with a probability of ≥ 0.25. From the induced genes in this dataset we performed co-expression analysis and found a total of 13 regulons with ≥ 4 nodes. Some of these regulons were enriched with functionally related genes among which parts of the previously known FIT target network and the iron homeostasis PYE-BTS regulon could be identified. The PYE-BTS regulon was almost completely present and could be extended by further genes.

Direct comparisons demonstrated large discrepancies between the proteomic and transcriptomic regulation [29] and remodeling the ribosomal composition has been proposed to cause biased translation [74]. The analysis of our virtual dataset appears to confirm such remodeling processes. However, the data in the virtual dataset display considerable overlap with combined proteomic data [42] at least at the functional level.

Taken together this study not only provides new insight into the effects of FIT abundance on gene expression but also points out the importance of redundant analyses.

## Methods

### Plant materials and plant growth

In this study we used the wild-type Arabidopsis ecotype Columbia-0 (Col-0) named WT, the *fit* knock-out line *fit-3* (GABI_108C10) [14] named *fit* and the FIT over-expressing line HA-FIT 8 [28] named HA-FIT. The seeds were sterilized and stratified for 48 h at 4 °C. Hydroponic growth was conducted as previously described using ¼-strength Hoagland medium without sucrose containing 10 μM iron [35]. The medium was exchanged every seven days. To prevent the *fit* plants from dying they were sprayed with Flory 72 (Fe-EDDHA) twice a week. After five weeks of hydroponic growth all plants were washed with ddH_2_O to rinse off residual Fe-EDDHA and the treatment was started by transferring the plants to fresh medium containing either 10 μM (+Fe) or 0 μM iron (-Fe). After seven days of treatment the six week-old plants were harvested. In the plate system stratified seeds were germinated in 12x12 cm^2^ square plates with 1 x Hoagland agar containing 50 μM (+Fe) or 0 μM iron (-Fe). After 6 days the seedlings were harvested.

### RNA extraction

One hundred milligrams of the roots of the six week-old hydroponically grown plants or 100 mg whole six day-old seedlings were frozen and homogenized under constant liquid nitrogen cooling, respectively. RNA extraction was performed with the RNEasy Plant Mini Kit (Qiagen) according to the manufacturer’s instructions. Total RNA content of the final extracts was measured fluorimetrically with the infinite M200PRO plate reader (TECAN) using the NanoQuant plate. RNA quality was estimated with the OD^260^/OD^280^ ratio.

### Microarray analysis

Two hundred nanogram of original total RNA were used per hybridization for the microarray analysis. The analysis was performed using CATMA microarrays. Three independent biological replicates were produced. For each biological replicate, RNA samples were prepared and analyzed in two technical replicates as previously described [29]. We analyzed gene expression in roots of six-week-old plants that were grown on  +Fe ¼-strength liquid Hoagland medium for five weeks and then transferred to  +Fe or -Fe for one week. We also analyzed gene expression in six-day-old whole seedlings that were grown on  +Fe or -Fe Hoagland agar for six days. Probes with a p value of ≤ 0.05 and a fold change of ≥1.5 were considered differentially expressed. The microarray data are publicly available at CATdb (http://urgv.evry.inra.fr/CATdb/; projects “AU15-01_Iron-FIT” and “AU13-06_FIT”). Microarray data from this article were deposited at Gene Expression Omnibus (http://www.ncbi.nlm.nih.gov/geo/), accession no. GSE65934 and GSE80281. The RNA preparations were also used for differential gene expression via RT-qPCR of selected genes identified in the microarray analysis (Additional file 2: Figure S2).

### Reverse transcription-quantitative polymerase chain reaction (RT-qPCR)

For RT-qPCR 1 μg of total RNA were treated with DNase. cDNA was synthesized using oligo-dT primers. The cDNA was diluted 1:10 with ddH_2_O, then once more 1:10 and 10 μl of this dilution were used per 20 μl PCR reaction. Using the DyNAmo ColorFlash SYBR Green qPCR Kit (Thermo Scientific) Real-time PCR was performed. A water negative control was treated equally. Quantification was based on mass standard curve analysis. Each sample value was normalized based on EF1Balpha2 expression. The average of 2 technical replicates was used as the sample expression value. The average of three biological replicates was calculated and ANOVA with Tukey’s HSD (Honestly Significant Difference) was performed for statistical analysis using the OriginPro 9.0 software. The primer sequences are shown in the Additional file 5: Table S8.

### Construction of the virtual dataset

For the construction of the virtual dataset we were interested in qualitative data, and for easier comparison with other experiments the expression data of previous publications as well as our own data were transformed so that in each comparison up-regulation was represented by the value 1 and down-regulation by the value -1. Below-threshold or insignificant regulation was given the value 0. We used our data and the provided expression data from 10 previous publications [11, 13, 19, 20, 24, 25, 63–65, 75]. From time course experiments we used the 24 h [24] or the 48 h and 72 h data [64], respectively. The reconstructed data from two publications [11, 20] must be considered incomplete since they only contain iron deficiency-induced genes. One dataset could not be reconstructed from the available supplementary information [75]. Together, 14 transcriptomic analyses from 9 studies have been taken into account (Additional file 3: Dataset 5).

From the transformed expression change values of the comparison WT -Fe vs. WT  +Fe we counted how often each gene was found regulated in any of the analyses irrespective of the direction of regulation. We abbreviated this value as “ABS” (absolute occurrence). Then we added the expression change values. We named the result “SUM” (sum of regulation values). If a gene was always or mostly regulated in one direction this resulted in a positive or negative value of SUM. We set the SUM threshold to ≥ 2 or ≤ -2 to ensure that a gene has been regulated at least twice more into one direction than into the other direction. Then we divided the absolute value of SUM by ABS to measure how often a contradictory regulation has been observed with the respective gene. We abbreviated this ratio as “RAT” (ratio between SUM and ABS). To ensure that the gene was regulated at least twice as often in one direction than into the other direction the threshold for RAT was set to ≥ 0.5. Only genes that matched the SUM and RAT thresholds were considered predominantly regulated into the respective direction under iron deficiency. All the other genes were considered regulated by other factors and removed from the dataset. To be able to rank the genes according to their uniformity of differential expression we introduced the “VIRT” value (virtual expected expression change) by dividing SUM (including the positive or negative sign) by the total number of analyses and multiplying the result with RAT. The total number of analyses was set to 14 for up-regulated genes (SUM > 2) since 14 analyses were used. For down-regulated genes (SUM < -2) we set the total number of analyses to 12 since 2 of the 14 analyses [11, 20] only contained genes that were induced under iron deficiency. The sign of VIRT indicates the direction of regulation and its absolute value roughly represents the probability to find the gene regulated into this direction. Genes that were found regulated in both directions got lower absolute VIRT values than genes that were found regulated in only one direction. We set the threshold of VIRT to be ≥ 0.25 or ≤ -0.25. Finally, the VIRT value was used to rank the genes in the virtual dataset according to their probability of regulation in the comparison WT -Fe vs. WT  +Fe (Additional file 3: Dataset 6).

## Additional files

Additional file 1: Figure S1. Overview and workflow of the analyses performed and the Arabidopsis lines used in this study. Three independent biological replicates of wild-type, HA-FIT and fit Arabidopsis plants were grown on iron-sufficient (+Fe) liquid medium for five weeks and then transferred to iron-sufficient or iron-deficient (-Fe) medium for one week (see also [29]). After a total of six weeks the roots were harvested. Seedlings were grown on iron-sufficient or iron-deficient Hoagland agar for six days and then the whole seedlings were harvested. From both, six-week-old roots and six-day-old seedlings, we extracted total RNA. This RNA was used to perform CATMA microarray analyses. RT-QPCR was performed to validate expression of a number of known iron homeostasis-related genes. By filtering the genes according to their expression patterns we were able to determine novel robustly FIT-induced and repressed genes and genes that were regulated in a FIT-dependent manner only in six-week-old roots or in six-day-old seedlings. Furthermore, we used our analyses plus previously published transcriptomic analyses to construct a virtual dataset with which we could determine robustly iron deficiency-regulated genes. (TIF 839 kb) Additional file 2: Figure S2. Validation of gene regulation by RT-qPCR analysis. Normalized absolute expression of the iron homeostasis-related genes FIT, IRT1, FRO2, AT3G07720, BHLH038, BHLH039, NAS1 and NAS2 (A-H) in six-day-old seedlings grown on iron-sufficient (+Fe) or iron-deficient (-Fe) medium. The horizontal point diagrams (I-P) indicate significant changes in the respective pairwise comparisons according to Tukey’s HSD. (TIF 1315 kb) Additional file 3: Supplemental Datasets.
**﻿Dataset 1.﻿ ** Fold-changes of gene expression in roots of 6 week-old Arabidopsis plants. The fold-changes are log_2_ values. In the column header the upper line/condition is compared to the lower line/condition. Significant up-regulation is indicated by red background color. Significant down-regulation is indicated by green background-color. The residual insignificant and below-threshold values are masked by black background color. **Dataset 2.** Fold-changes of gene expression in 6 day-old Arabidopsis whole seedlings. The fold-changes are log_2_ values. In the column header the upper line/condition is compared to the lower line/condition. Significant up-regulation is indicated by red background color. Significant down-regulation is indicated by green background-color. The residual insignificant and below-threshold values are masked by black background color. **Dataset 3.** Juxtaposition of the fold-changes of gene expression in roots of 6-day-old Arabidopsis seedlings and 6-week-old Arabidopsis roots. The fold-changes are log_2_ values. In the column header the upper line/condition is compared to the lower line/condition. Significant up-regulation is indicated by red background color. Significant down-regulation is indicated by green background-color. The residual insignificant and below-threshold values are masked by black background color. **Dataset 4.** Juxtaposition of the fold-changes of gene expression in roots of 6-day-old Arabidopsis seedlings and 6-week-old Arabidopsis roots. The fold-changes are log_2_ values. In the column header the upper line/condition is compared to the lower line/condition. Significant up-regulation is indicated by red background color. Significant down-regulation is indicated by green background-color. The residual insignificant and below-threshold values are masked by black background color. The genes were filtered so that only genes are present in this table which were found regulated at least in one comparison in whole seedlings and in one comparison in roots. **Dataset 5:** Genes that are regulated in an iron-dependent manner across 10 studies with a total of 14 comparisons. Up-regulated genes are represented by the value 1 and a red background color. Down-regulated genes are represented by the value -1 and a green background color. Genes with no significant regulation or with regulation below the threshold of the respective study are represented by the value 0 and black background color. The column titled ‘ABS’ shows the number of comparisons in which the gene was found regulated. The column titled ‘SUM’ shows the overall sum of the given values (-1 for down-regulation, +1 for up-regulation, ±0 for no regulation) of all comparisons. The genes with the highest sum are regarded as most stably up-regulated upon iron deficiency. The genes with the lowest sum are regarded as most stably down-regulated upon iron deficiency. The column titled ‘RAT’ contains the result of division of the absolute value of ‘SUM’ by ‘ABS’ and is supposed to provide a threshold for the Supplemental Dataset 6, along with ‘SUM’. (XLSX 4180 kb) Additional file 4: Figure S3. Close view of the iron homeostasis cluster in six-day-old seedlings and six-week-old roots as shown in Fig. 2d. The indicator genes and their respective expression patterns are highlighted yellow. (TIF 71 kb) Additional file 5: Supplemental Tables.
**Table S1.** List of genes that were found regulated in the inter-line and intra-line comparisons in six-day-old seedlings and roots of six-week-old plants and that were found in iron homeostasis-related clusters after hierarchical clustering of the seedling, root and combined expression data. **Table S2.** Lists of input genes and the output genes before and after each of the four filtering steps. The lists are sorted alphabetically. **Table S3.** Expression patterns of genes that are induced by FIT in six-day-old seedlings. The genes marked red were found regulated in seedlings but not in the root samples. The genes marked yellow have previously been associated with iron homeostasis. For comparison, the respective root expression patterns have been added to the right. Additionally, we have added a column (S) with selected publications in which mutants have been investigated or which establish a direct link to iron homeostasis. **Table S4.** Expression patterns of genes that are induced by FIT in roots of six-week-old plants. The genes marked red were found regulated in the root samples but not in seedlings. For comparison the respective seedling expression patterns have been added to the right. **Table S5.** Expression patterns of genes that are repressed by FIT in six-week-old roots. The genes marked red were found regulated in the root samples but not in seedlings. For comparison the respective seedling expression patterns have been added to the right. **Table S6.** Expression patterns of genes that are repressed by FIT in six-day-old seedlings. The genes marked red were found regulated in seedlings but not in the root samples. For comparison the respective seedling expression patterns have been added to the right. **Table S7.** List of genes that were found in common regulons with ≥ 4 nodes in the co-expression analysis of iron deficiency-induced genes in the virtual dataset. The numbers correspond to the numbering in Fig. 7. **Table S8.** List of primers which we used to validate the expression data of some iron homeostasis-related genes. (XLSX 197 kb) Additional file 6: Figure S4. Co-expression network built from the genes induced under -Fe in the virtual dataset: Regulon 1: contains members of the FIT target network [13]. Regulon 2: consists of members of the PYE-BTS regulon [13]. Regulon 3: is largely composed of genes involved in phenylpropanoid metabolism. Regulon 4: mainly comprises genes that participate in the pentose phosphate pathway, glycolysis and gluconeogenesis. Regulon 5: is mostly composed of genes that are involved in RNA processing and translation. Regulon 6: contains mitochondrial proteins. Regulon 7: is heterogeneous but contains comparably many chaperons. Regulon 8: is enriched in genes involved in amino acid metabolism. Regulon 9: is also heterogeneous but enriched in genes that participate in plant-pathogen interaction. Regulon 10: shows no enrichment of molecular functions. Regulon 11: mainly contains genes that participate in purine, lipid and aromatic compound metabolism. Regulon 12: is composed of genes involved in the response to low sulfur. Regulon 13 shows no enrichment of molecular functions. The network has been created with the String version 10 protein interaction database [27]. The confidence was set to ‘medium’ (0.400) and no genes were added. The 437 genes induced under -Fe in the virtual dataset were used as input. Singlet nodes have been removed and only networks with 4 or more nodes are shown. The resulting network image contains 169 genes (Additional file 5: Table S7). (TIF 8234 kb)

## Abbreviations

CATMA: Complete Arabidopsis Transcriptome MicroArray
RT-qPCR: Reverse transcription quantitative polymerase chain reaction
WT: wild-type
RNA-seq: RNA sequencing
JA: Jasmonic acid
SA: Salicylic acid
IAA: Indole-3-acetic acid
MeIAA: Methyl-indole-3-acetic acid
EDDHA: Ethylenediamine-N,N'-bis(2-hydroxyphenylacetic acid)
ANOVA: Analysis of variance
HSD: Honestly Significant Difference
