# Supplementary figures and images for: Iron homeostasis in Arabidopsis thaliana: transcriptomic analyses reveal novel FIT-regulated genes, iron deficiency marker genes and functional gene networks
Source: BMC Plant Biol. 2016 Oct 3;16:211. doi: 10.1186/s12870-016-0899-9 (PMC5048462; doi:10.1186/s12870-016-0899-9)

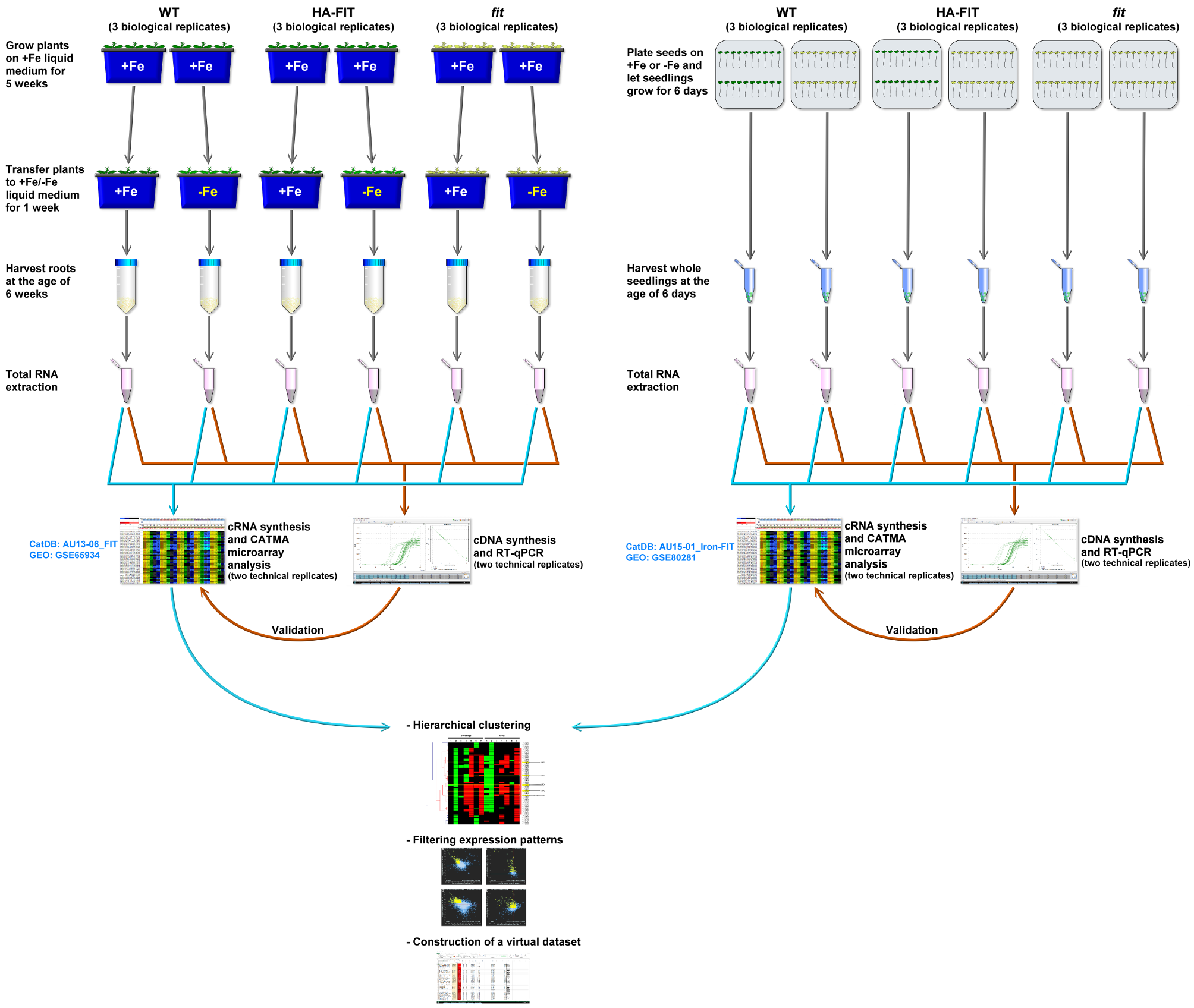

Supplement: Additional file 1: Figure S1. — Overview and workflow of the analyses performed and the Arabidopsis lines used in this study. Three independent biological replicates of wild-type, HA-FIT and fit Arabidopsis plants were grown on iron-sufficient (+Fe) liquid medium for five weeks and then transferred to iron-sufficient or iron-deficient (-Fe) medium for one week (see also [29]). After a total of six weeks the roots were harvested. Seedlings were grown on iron-sufficient or iron-deficient Hoagland agar for six days and then the whole seedlings were harvested. From both, six-week-old roots and six-day-old seedlings, we extracted total RNA. This RNA was used to perform CATMA microarray analyses. RT-QPCR was performed to validate expression of a number of known iron homeostasis-related genes. By filtering the genes according to their expression patterns we were able to determine novel robustly FIT-induced and repressed genes and genes that were regulated in a FIT-dependent manner only in six-week-old roots or in six-day-old seedlings. Furthermore, we used our analyses plus previously published transcriptomic analyses to construct a virtual dataset with which we could determine robustly iron deficiency-regulated genes. (TIF 839 kb) [file 12870_2016_899_MOESM1_ESM.tif]

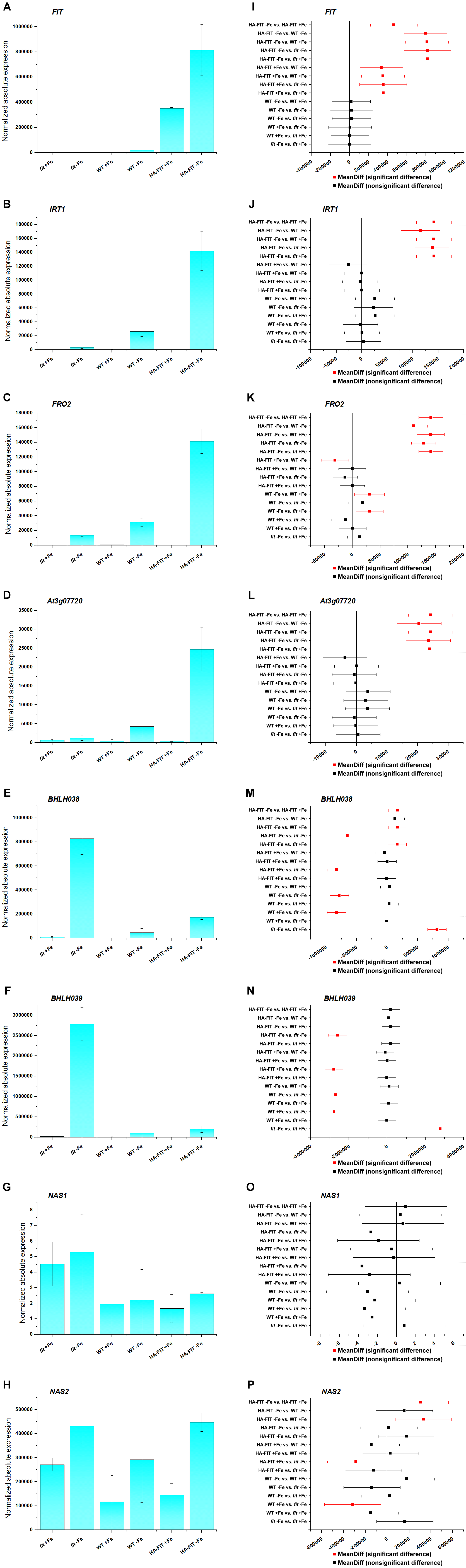

Supplement: Additional file 2: Figure S2. — Validation of gene regulation by RT-qPCR analysis. Normalized absolute expression of the iron homeostasis-related genes FIT, IRT1, FRO2, AT3G07720, BHLH038, BHLH039, NAS1 and NAS2 (A-H) in six-day-old seedlings grown on iron-sufficient (+Fe) or iron-deficient (-Fe) medium. The horizontal point diagrams (I-P) indicate significant changes in the respective pairwise comparisons according to Tukey’s HSD. (TIF 1315 kb) [file 12870_2016_899_MOESM2_ESM.tif]

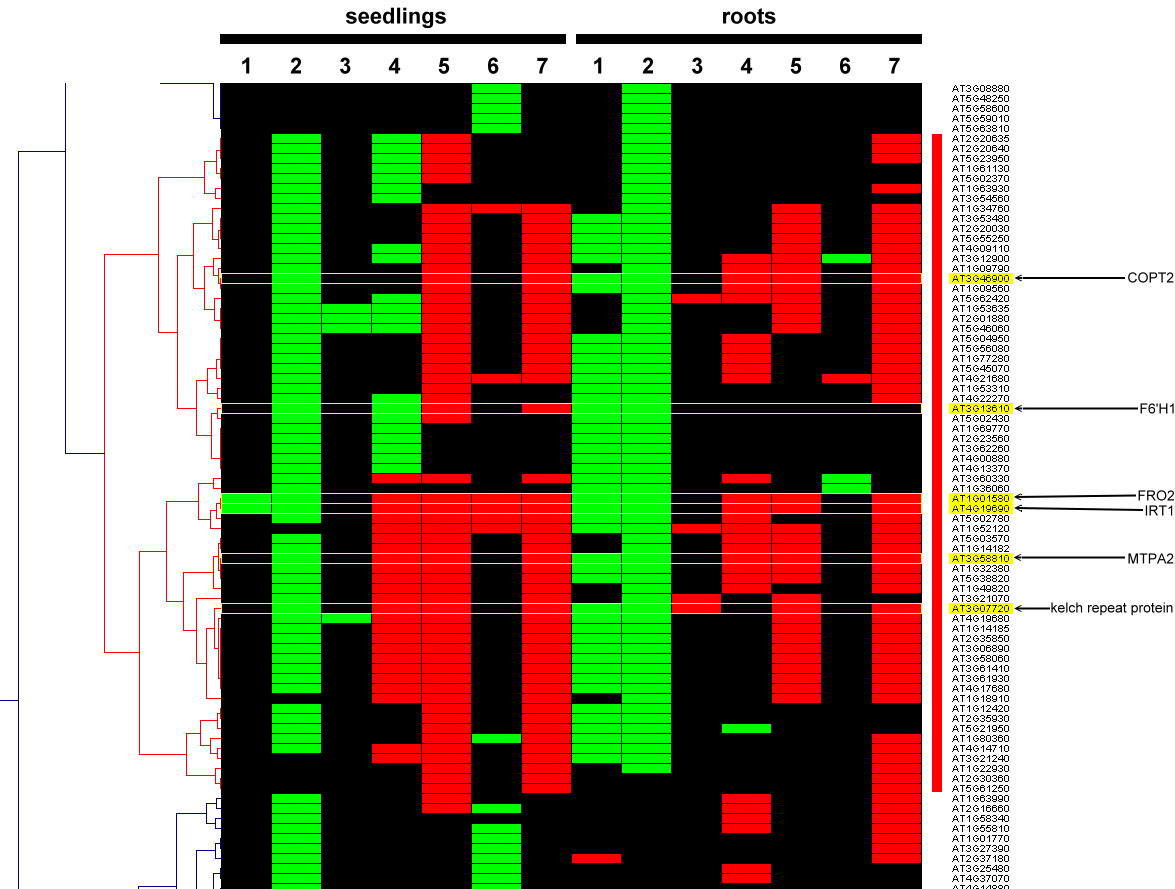

Supplement: Additional file 4: Figure S3. — Close view of the iron homeostasis cluster in six-day-old seedlings and six-week-old roots as shown in Fig. 2d. The indicator genes and their respective expression patterns are highlighted yellow. (TIF 71 kb) [file 12870_2016_899_MOESM4_ESM.tif]

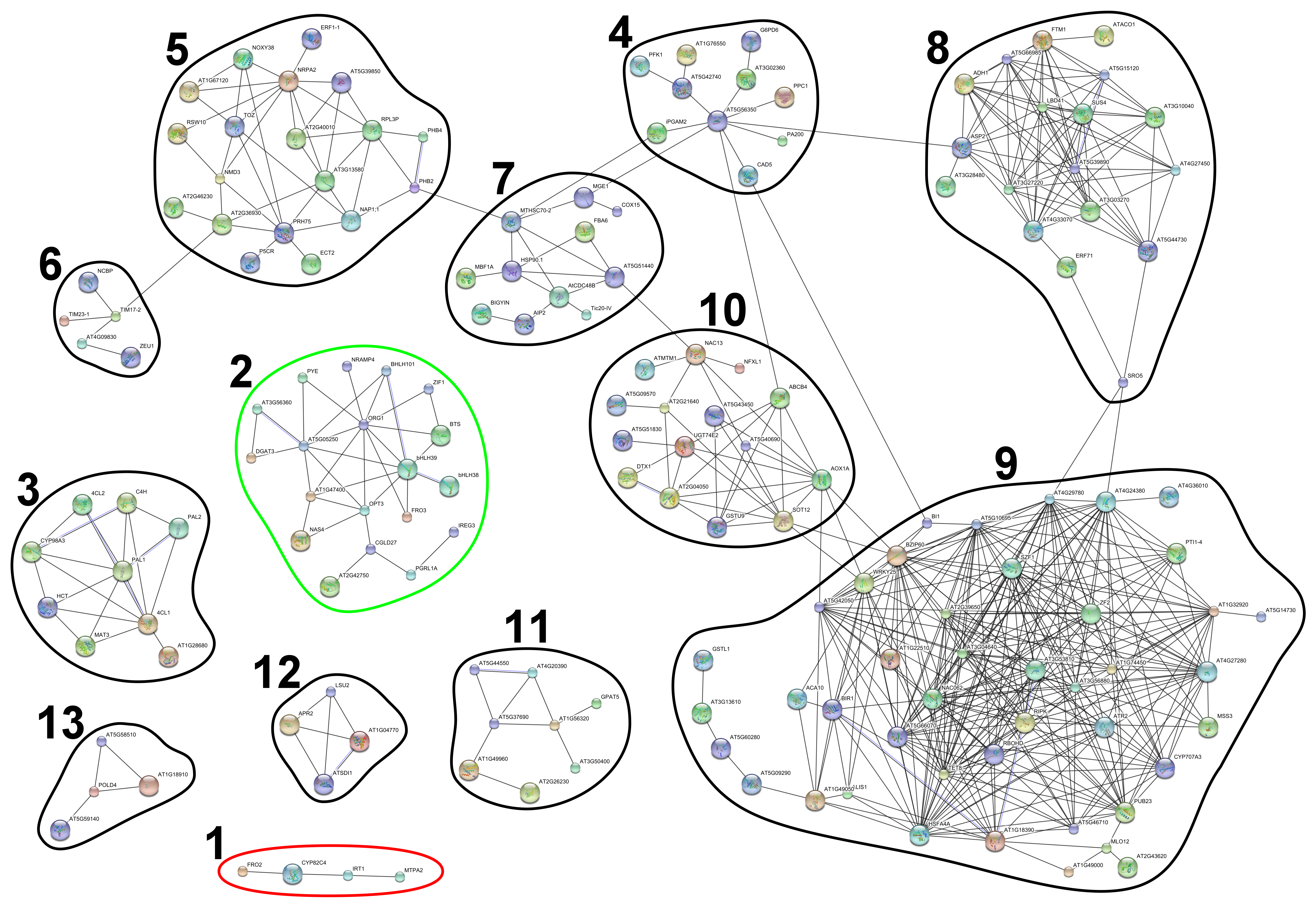

Supplement: Additional file 6: Figure S4. — Co-expression network built from the genes induced under -Fe in the virtual dataset: Regulon 1: contains members of the FIT target network [13]. Regulon 2: consists of members of the PYE-BTS regulon [13]. Regulon 3: is largely composed of genes involved in phenylpropanoid metabolism. Regulon 4: mainly comprises genes that participate in the pentose phosphate pathway, glycolysis and gluconeogenesis. Regulon 5: is mostly composed of genes that are involved in RNA processing and translation. Regulon 6: contains mitochondrial proteins. Regulon 7: is heterogeneous but contains comparably many chaperons. Regulon 8: is enriched in genes involved in amino acid metabolism. Regulon 9: is also heterogeneous but enriched in genes that participate in plant-pathogen interaction. Regulon 10: shows no enrichment of molecular functions. Regulon 11: mainly contains genes that participate in purine, lipid and aromatic compound metabolism. Regulon 12: is composed of genes involved in the response to low sulfur. Regulon 13 shows no enrichment of molecular functions. The network has been created with the String version 10 protein interaction database [27]. The confidence was set to ‘medium’ (0.400) and no genes were added. The 437 genes induced under -Fe in the virtual dataset were used as input. Singlet nodes have been removed and only networks with 4 or more nodes are shown. The resulting network image contains 169 genes (Additional file 5: Table S7). (TIF 8234 kb) [file 12870_2016_899_MOESM6_ESM.tif]
